# Linear Polymer Cathode Materials for Highly Efficient Aqueous Zinc‐Ion Batteries: Is the High Active Sites Density Necessary?

**DOI:** 10.1002/advs.202503156

**Published:** 2025-04-26

**Authors:** Yiyang Dai, Yao Yao, Liang Feng, Zhenglong Qiu, Min Deng, Qiang Peng

**Affiliations:** ^1^ College of Materials and Chemistry & Chemical Engineering Chengdu University of Technology Chengdu 610059 P. R. China; ^2^ School of Chemical Engineering and State Key Laboratory of Polymer Materials Engineering Sichuan University Chengdu 610065 P. R. China

**Keywords:** active site density, aqueous zinc ion batteries, energy storage performance, polymer cathode materials

## Abstract

Recently, research on aqueous zinc‐ion batteries (AZIBs) has always focused on improving the energy storage performance by increasing the number of active sites, particularly in designing organic/polymer materials with a high density of active sites. However, does a higher density of active sites necessarily induce enhanced energy storage performance? To verify this issue, we have designed two linear polymers, where TAPT‐DHBQ contains an additional pair of active sites (carbonyl groups) compared to TABQ‐DHBQ, with theoretical specific capacities of 545.26 and 379.14 mAh g^−1^, respectively. Interestingly, the experimental results have deviated with the specific capacities of these polymers being comparable, measuring to be 325 mAh g^−1^ (TABQ‐DHBQ) and 280 mAh g^−1^ (TAPT‐DHBQ). This is attributed to the competition effect between neighboring active sites, which leads to decreased utilization of active sites. As a result, the Zn//TABQ‐DHBQ batteries with ZnI_2_ electrolyte additive have exhibited high specific capacities of 618 and 360 mAh g^−1^ at the current densities of 1 and 10 A g^−1^, along with a high energy density of 678.6 Wh kg^−1^ (1 A g^−1^). The finding underscores the importance of uniform electron cloud distribution in cathode materials for achieving efficient AZIBs.

## Graphical abstract

Two linear polymers with uniformly distributed electron clouds to verify the necessary of high‐density active sites, which enable extremely high active site utilization of 85.7%, thus resulting in superb high specific capacity of 618 mAh g^−1^ at 1 A g^−1^ and energy density of 678.62 Wh kg^−1^ in ZnSO_4_ and ZnI_2_ mixture electrolyte.

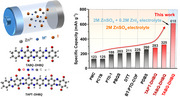

## 1 Introduction

In recent years, aqueous zinc‐ion batteries (AZIBs) have attracted significant attention due to their potential as substitutes for lithium‐ion batteries, owing to their desirable theoretical specific capacity, low redox potential, high safety, low cost, sustainability, and environmental friendliness.^[^ ^1^, ^2^, ^3^, ^4^, ^5^ ^]^ However, the commercialization of AZIBs is hindered by their insufficient energy density.^[^ ^1^ ^]^ To address this issue, two main solutions have been proposed: one involves the development of cathodes with high specific capacity,^[^ ^6^ ^]^ while the other focuses on enhancing the discharge potential platform of AZIBs.^[^ ^7^, ^8^ ^]^ To effectively enhance the energy density of AZIBs, researchers have explored various electrolyte additives. Among these additives, the redox reaction involving iodide and iodine (I₂ + 2e⁻ ↔ 2I⁻; I₃⁻ + 2e⁻ ↔ 3I⁻) offers a high redox potential and facilitates multiple charge‐transfer processes. The introduction of iodide additives into AZIB systems has been shown to promote the reversible coordination of Zn^2+^/H^+^, thereby improving the discharge plateau and specific capacity, and ultimately enhancing the energy density of AZIBs.^[^ ^9^, ^10^, ^11^, ^12^ ^]^ However, due to the “shuttle effect” caused by the migration of polyiodide species generated during the redox process, this additive often leads to continuous loss of active materials, necessitating further improvements to enhance its stability.^[^ ^14^ ^]^ On the other hand, previously developed cathodes have been mainly based on inorganic compounds, such as manganese (Mn)‐ and vanadium (V)‐based compounds, as well as Prussian blue (PB) analogs.^[^ ^14^, ^15^, ^16^ ^]^ These cathodes have reversibly achieved Zn^2+^/H^+^ storage through the transformation of crystal structures and elemental valence states during the charge/discharge process.^[^ ^17^, ^18^, ^19^ ^]^ Nevertheless, the lattice of inorganic cathodes often experiences significant volume expansion and even structural collapse during the charge/discharge process, thus leading to rapid decay in specific capacity and poor long‐term cycle stability.^[^ ^20^, ^21^, ^22^ ^]^ Moreover, these inorganic cathodes typically exhibit poor electrical conductivity, which contributes to sluggish kinetics and unsatisfactory rate performance.^[^ ^23^, ^24^, ^25^ ^]^

Compared to inorganic cathodes, organic compounds have gradually gained attention because of their structural designability, lattice flexibility, and resource renewability.^[^ ^26^, ^27^, ^28^ ^]^ Among them, although small organic molecules exhibit high specific capacity, their poor long‐term cycle stability is unsatisfactory because they often dissolve easily in aqueous electrolytes, such as TABQ, C4Q, tetrachloro‐1,4 benzoquinone, and π‐PMC.^[^ ^29^, ^30^, ^31^, ^32^, ^33^ ^]^ Thus, polymers have been designed and synthesized, including linear polymers,^[^ ^34^, ^35^, ^36^, ^37^ ^]^ porous polymers,^[^ ^38^, ^39^ ^]^ and covalent organic frameworks.^[^ ^6^, ^40^, ^41^, ^42^ ^]^ In these materials, it has been found that carbonyl and imine functional groups can serve as effective active sites for storing Zn^2+^/H^+^, known as n‐type organic cathodes.^[^ ^22^, ^43^, ^44^ ^]^ In fact, organic small molecules containing carbonyl and imine groups are commonly utilized as monomers to synthesize porous polymers or covalent organic frameworks, while other organic small molecules without any energy storage active site are employed as linkers to construct porous structures.^[^ ^45^, ^46^ ^]^ This design approach introduces a large number of functional groups that are ineffective for energy storage, thereby leading to a limited specific capacity.^[^ ^47^ ^]^ Moreover, the preparation of these materials typically involves high temperatures, harsh conditions, and prolonged processing times, making the process complex with a low success rate and high cost.^[^ ^48^ ^]^ In contrast, linear polymers are not limited by linkers, allowing small organic molecules with enough active sites to be selected as monomers. Additionally, linear polymers are simpler to prepare with a high success rate and cost‐effectiveness, making them more suitable for large‐scale synthesis.^[^ ^49^ ^]^ Based on this finding, researchers prefer designing linear polymers with a high density of carbonyl and imine functional groups to enhance the specific capacity of AZIBs cathodes.^[^ ^41^, ^42^, ^50^, ^51^ ^]^ However, this will bring a new question. Does a higher density of energy storage active sites always result in better performance?

**Figure 1:**
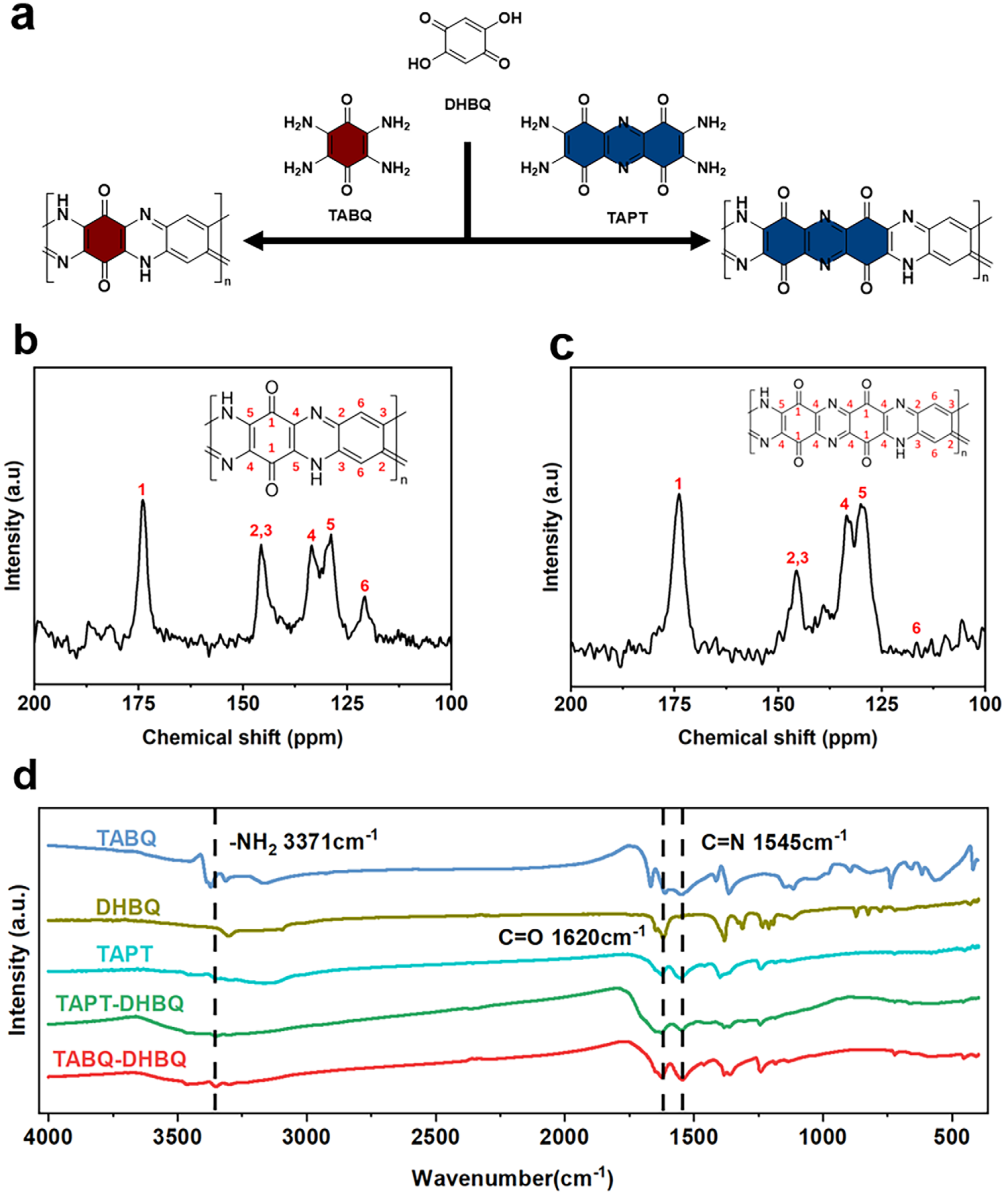
a) The synthetic routes of TABQ‐DHBQ and TAPT‐DHBQ. b, c) The ^13^C solid‐state NMR spectra of TABQ‐DHBQ (b) and TAPT‐DHBQ (c). d) The FTIR spectra of TABQ‐DHBQ, TAPT‐DHBQ, and monomers.

In this work, we have designed and synthesized two linear polymers (namely TABQ‐DHBQ and TAPT‐DHBQ) via a one‐step polycondensation reaction using 2,3,5,6‐tetraaminocyclohexa‐2,5‐diene‐1,4‐dione (TABQ), 2,3,7,8‐tetraaminophenazine‐1,4,6,9‐tetraone (TAPT) and 2,5‐dihydroxycyclohexa‐2,5‐diene‐1,4‐dione (DHBQ) as monomers (**Figure** **1**a). These two polymers form a highly ordered 2D structure through alternating conjugation of benzene and heterocyclic rings, with multiple C═O and C═N active energy storage functional groups evenly distributed along the polymer chains to facilitate the reversible coordination of Zn^2+^/H^+^. In contrast, TAPT‐DHBQ contains an additional pair of C═O groups compared to TABQ‐DHBQ, thereby leading to a higher density of active sites. The theoretical specific capacity of TAPT‐DHBQ (545.26 mAh g^−1^) is significantly higher than that of TABQ‐DHBQ (379.14 mAh g^−1^). However, experimental results have indicated that TAPT‐DHBQ has exhibited a specific capacity of only 280 mAh g^−1^, which is lower than that of TABQ‐DHBQ (325 mAh g^−1^). To explain this phenomenon, we have performed density functional theory (DFT) calculations, which show that the high density of energy‐storage active sites in TAPT‐DHBQ leads to competitive interactions, resulting in the dispersed negative electron cloud around the active sites. This reduced redox reactivity toward coordination with Zn^2+^/H^+^ can contribute to the low utilization of the active sites. This finding underscores the importance of electrochemical conditions surrounding the active sites. To further enhance the commercialization potential of these AZIBs, the ZnI_2_ electrolyte additive has been introduced. The introduced iodide can be effectively adsorbed onto the surfaces of the two polymers, reducing the charge transfer resistance (R_ct_) at the electrode/electrolyte interface and promoting the reversible coordination of Zn^2+^/H^+^. Simultaneously, the adsorption of iodide onto the polymer skeletons enhances the electrical conductivities of both polymers, facilitating electron transfer and thereby supporting the rapid charge/discharge process of AZIBs at high current densities while ensuring superior rate performance. Furthermore, the introduction of iodide inevitably triggers redox reactions, contributing to the specific capacities and improving the discharge plateau of AZIBs. Benefit from the synergistic effect of iodide, as a result, the energy storage performance of TABQ‐DHBQ cathode has been improved to 618 mAh g^−1^ at a current density of 1 A g^−1^ with an energy density of 678.62 Wh kg^−1^, while the specific capacity of TAPT‐DHBQ cathode has been enhanced to 607 mAh g^−1^ at a current density of 1 A g^−1^ along with an energy density of 670.7 Wh kg^−1^. Simultaneously, DFT calculations reveal that both polymers exhibit a strong adsorption affinity for triiodine ions, effectively immobilizing polyiodide species and mitigating their shuttle effect. Consequently, with the incorporation of ZnI_2_ additives, these two polymers demonstrate exceptional long‐term cycling stability. Even after 2000 cycles at a current density of 10 A g^−1^, the specific capacity remains at 63.2% (TABQ‐DHBQ) and 63.3% (TAPT‐DHBQ), with ≈100% coulombic efficiency (CE). This work provides a new insight into the rational design of polymer cathodes for high‐performance AZIBs. When designing high‐performance organic or polymer cathodes of AZIBs, more attention should be given to the electrochemical environment surrounding the active sites, rather than merely increasing the number of active sites.

## 2 Results and Discussion

**Figure 2:**
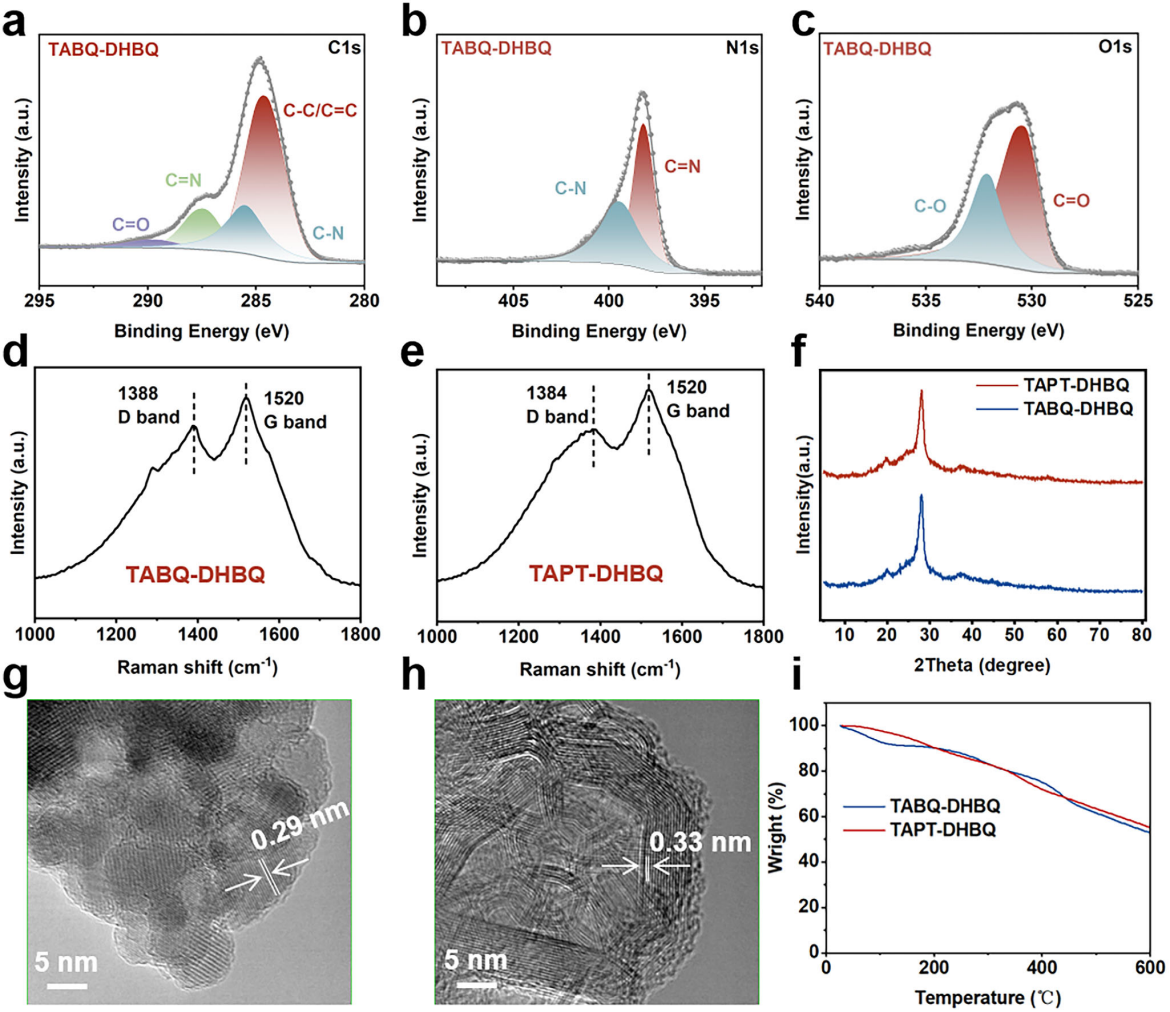
a–c) High‐resolution C1s (a), N1s (b), and O1s (c) XPS spectra of TABQ‐DHBQ polymer. d, e) Raman spectra of TABQ‐DHBQ (d) and TAPT‐DHBA polymers (e). f) XRD patterns of TABQ‐DHBQ and TAPT‐DHBQ polymers. g, h) HRTEM images of TABQ‐DHBQ (g) and TAPT‐DHBQ (h) polymers. i) TGA curves of TABQ‐DHBQ and TAPT‐DHBQ polymers.

TABQ‐DHBQ and TAPT‐DHBQ linear polymers were synthesized via a one‐step polycondensation reaction by using DHBQ and TABQ (or TAPT) as monomers (Figure 1a). The molecular structures of these polymers were characterized by Fourier transform infrared (FTIR) spectroscopy and ^13^C solid‐state nuclear magnetic resonance (NMR) spectroscopy (Figure 1b–d). The ^13^C solid‐state NMR spectra (Figure 1b,c) identified six distinct types of carbon atoms (e.g., C═O, C═N, C‐C/C═C, C═C‐N‐H, C═C‐N, and O═C‐C‐N), labeled in the inset structures of these two polymers. In particular, the overlapping peaks corresponding to the π‐conjugated structure would result in broadened characteristic peaks in the solid‐state NMR spectra.^[^ ^52^, ^53^ ^]^ Subsequently, FTIR spectra were performed to further characterize their structure. As depicted in Figure 1d, distinct characteristic peaks were observed in the FTIR spectra of both polymers at 1620 and 1545 cm^−1^, corresponding to the stretching vibrations of the C═N and C═O bonds.^[^ ^40^, ^54^ ^]^ Notably, the characteristic peak at 3371 cm^−1^ was attributed to the ‐NH_2_ group in the FTIR spectra of TABQ and TAPT monomers.^[^ ^21^ ^]^ This peak was notably weakened or even completely disappeared in the FTIR spectra of polymers, confirming the successful completion of the polycondensation reaction. In addition to these measurements, the chemical structures of these two polymers were further characterized by X‐ray photoelectron spectroscopy (XPS) (**Figure** **2**a–c; Figures S1 and S2, Supporting Information). Specifically, the presence of ‐C‐N (400 eV) and ‐C═N (398 eV) in the high‐resolution N1s XPS spectra (Figure 2b) confirmed the structure of TABQ‐DHBQ polymer, which corroborated by the high‐resolution C1s and O1s XPS spectra (Figure 2a,c). Figure S2 (Supporting Information) further demonstrates the structure of TAPT‐DHBQ polymer. The solubility of both polymers in common organic solvents was extremely low, including 1,2,4‐trichlorobenzene, tetrahydrofuran (THF), N,N‐dimethylformamide (DMF) and dimethyl sulfoxide (DMSO). As shown in Figure S3 (Supporting Information), the molecular weight of the fraction that was slightly soluble in 1,2,4‐trichlorobenzene was determined by gel permeation chromatography (GPC) to be ≈8000 Da (TAPT‐DHBQ) and 9500 Da (TABQ‐DHBQ), indicating a high degree of polymerization. Based on this, the 2D structure of the two polymers was further analyzed by X‐ray diffraction (XRD), Raman spectroscopy, and high‐resolution transmission electron microscope (HRTEM). The Raman spectra of both polymers (Figure 2d,e) exhibited a distinct G‐band characteristic peak at ≈1580 cm^−1^, attributed to the in‐plane vibration of sp^2^‐hybridized carbon atoms, confirming the presence of an extended planar π‐conjugated structure.^[^ ^55^ ^]^ XRD measurements were conducted, revealing a distinct peak at 26° in the patterns of both polymers (Figure 2f), also indicative of π‐π stacking interactions within the conjugated structures.^[^ ^45^ ^]^ The larger planar π‐π conjugated system typically exhibited high electrical conductivity. Moreover, a sharp diffraction peak appeared at 28° in the XRD patterns of both polymers. Calculations revealed that the full widths at half maximum (FWHM) of the diffraction peaks were 4.45 for TABQ‐DHBQ polymer and 8.86 for TAPT‐DHBQ polymer, indicating that TABQ‐DHBQ polymer exhibited a more ordered layer‐by‐layer stacking structure than TAPT‐DHBQ polymer. HRTEM images (Figure 2g,h; Figure S4, Supporting Information) also displayed distinct lattice fringes, with interlayer spacings of 0.29 nm (TABQ‐DHBQ) and 0.33 nm (TAPT‐DHBQ), further confirming their 2D layer‐by‐layer stacking structure. The interlayer spacing, which was significantly larger than the radius of Zn^2+^ (0.074 nm), provided efficient ion transport channels, enabling rapid ion migration and improving reaction kinetics.^[^ ^56^ ^]^ Furthermore, the 2D structure facilitated the exposure of high‐density active sites to a maximum extent, thereby enhancing their utilization. The morphologies of these two polymers were studied by scanning electron microscopy (SEM) images (Figure S5, Supporting Information). Both polymers clearly exhibited bulk‐like microstructures. Moreover, energy‐dispersive X‐ray spectroscopy (EDS) elemental mappings indicated a homogeneous distribution of C, N, and O elements across both polymer materials (Figure S6, Supporting Information). The thermal stability of the polymers was evaluated by thermogravimetric analysis (TGA) under nitrogen. As shown in Figure 2i, a significant mass loss was observed at temperatures above 300 °C, indicating their high thermal stability. Furthermore, the stability in aqueous electrolytes was investigated by UV–vis spectra (Figure S7, Supporting Information). The UV–vis spectra of the ZnSO_4_ aqueous electrolyte after electrode immersion closely matched those of the pristine electrolyte even after 72 hours, confirming the insolubility of two polymers in the ZnSO_4_ aqueous electrolyte.

**Figure 3:**
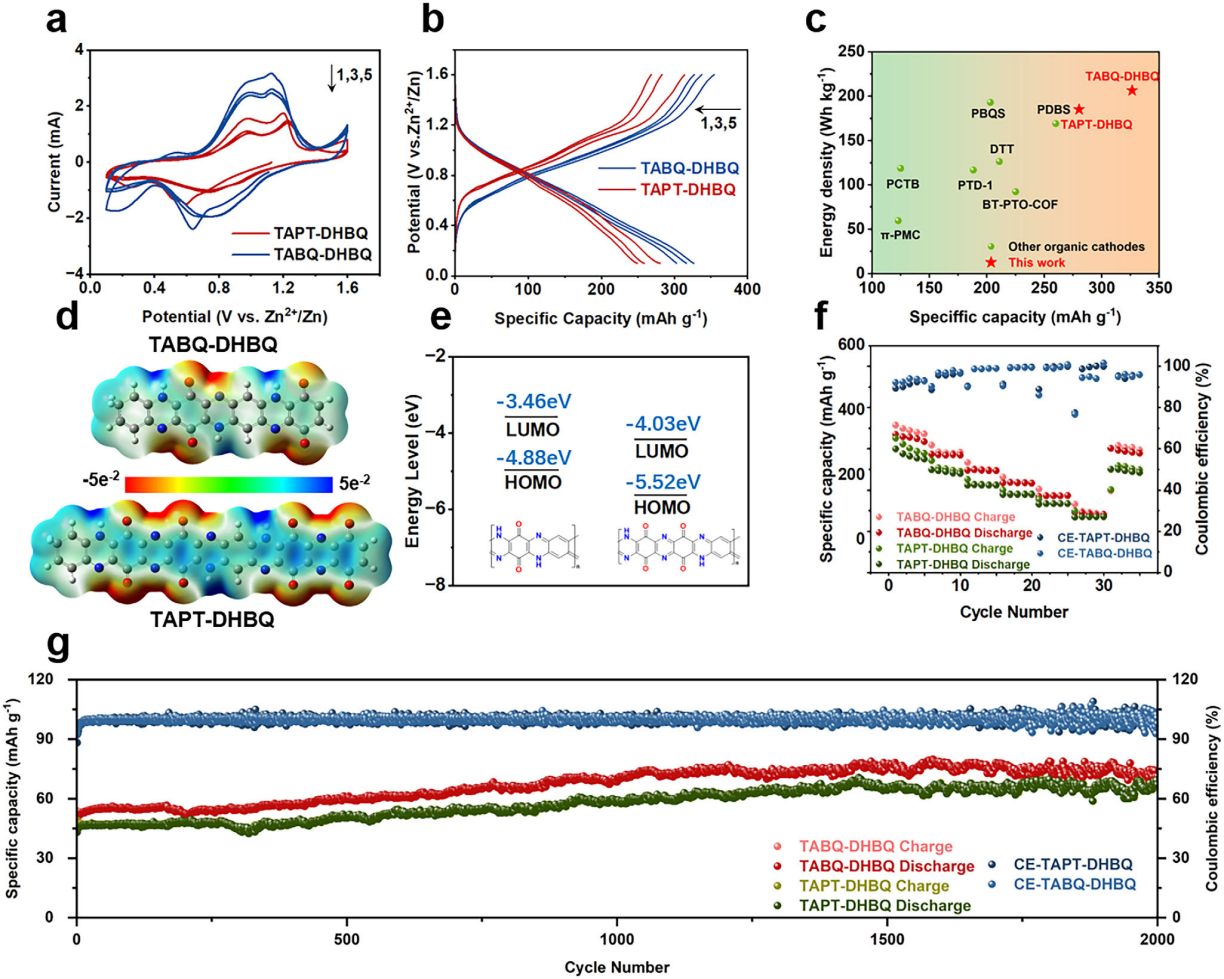
a) CV curves of TABQ‐DHBQ and TAPT‐DHBQ cathodes at a scan rate of 1 mV s^−1^. b) GCD curves of TABQ‐DHBQ and TAPT‐DHBQ cathodes at a current density of 0.1 A g^−1^. c) Rate performance of TABQ‐DHBQ and TAPT‐DHBQ cathodes. d) Electronic static potential (ESP) mappings of TABQ‐DHBQ and TAPT‐DHBQ polymers. e) HOMO and LUMO energy levels and energy gaps of TABQ‐DHBQ and TAPT‐DHBQ polymers. f) Energy storage performance comparison with other organic cathodes reported previously.^[^ ^32^, ^33^, ^45^, ^56^, ^57^, ^58^, ^59^ ^]^ g) Long‐term cycling stability of TABQ‐DHBQ and TAPT‐DHBQ cathodes carried out at the current density of 5 A g^−1^.

To evaluate the electrochemical performance of these polymers, we assembled 2032‐coin cells with these two polymers used as cathodes, along with zinc foil as anode and 2 m ZnSO_4_ aqueous solution as an electrolyte, respectively. The cyclic voltammetry (CV) curves of AZIBs based on the resulting polymers, shown in **Figure** **3**a, clearly exhibited two pairs of redox peaks. The oxidation peak potentials of the TABQ‐DHBQ polymer were observed at 0.85 and 1.13 V, while the reduction peak potentials were observed at 0.66 and 0.84 V, respectively. For the TAPT‐DHBQ polymer, the oxidation peak potentials appeared at 0.90 and 1.17 V, with the reduction peak potentials at 0.56 and 0.80 V, respectively. These redox peaks corresponded to the Zn^2+^/H^+^ storage processes at the C═O and C═N active sites, respectively.^[^ ^54^ ^]^ Specifically, the 0.85/0.66 V redox peaks of TABQ‐DHBQ polymer and 0.90/0.56 V redox peaks of TAPT‐DHBQ polymer corresponded to the revisable coordination between C═O active sites and Zn^2+^/H^+^. Meanwhile, the 1.13/0.84 V redox peaks of TABQ‐DHBQ polymer and 1.17/0.80 V redox peaks of TAPT‐DHBQ polymer corresponded to the revisable coordination between C═N active sites and Zn^2+^/H^+^, respectively.^[^ ^57^ ^]^ With increasing the number of CV cycles, the redox peak currents exhibited variations, suggesting that the energy storage process required an activation period during the initial cycles.^[^ ^41^ ^]^ This observation was further supported by electrochemical impedance spectroscopy (EIS) measurements (Figure S8, Supporting Information), which showed a significant decrease in charge transfer impedance (R_ct_), followed by a slight increase and eventual stabilization during the charge/discharge processes. This behavior further indicated a brief activation process during the initial stages of energy storage.^[^ ^55^ ^]^ In addition, a distinct oxidation peak was observed at 0.51 V during the first cycle of CV curves for TABQ‐DHBQ cathode, which subsequently disappeared. This phenomenon was primarily attributed to the transformation of the C‐N bond in TABQ‐DHBQ polymer to the C═N bond during the first charging process. Moreover, the integrated areas under the CV curves of the polymers were similar, suggesting their comparable electrochemical performance. The galvanostatic charge/discharge (GCD) tests also supported these results, as shown in Figure 3b. The AZIBs based on TABQ‐DHBQ and TAPT‐DHBQ cathodes exhibited initial specific capacity of 325^1^ and 280 mAh g^−1^ (0.1 A g^−1^), respectively, which remained at 303 and 249 mAh g^−1^ after five charge/discharge cycles. Subsequently, the discharge platforms were determined from the dQ/dV curves (Figure S9, Supporting Information). The two discharge platforms of TABQ‐DHBQ were 0.53–0.86 V and 0.86–1.36 V, while those of TAPT‐DHBQ were 0.41–0.72 V and 0.72–1.28 V, which corresponded to the CV redox peaks of the two polymers. Based on this, the energy densities of AZIBs based on these two polymer cathodes were calculated to be 206 Wh kg^−1^ (TABQ‐DHBQ) and 185 Wh kg^−1^ (TAPT‐DHBQ), respectively. These values were relatively higher than those of other linear polymer cathodes to date (Figure 3c; Table S1, Supporting Information).^[^ ^32^, ^33^, ^45^, ^56^, ^57^, ^58^, ^59^ ^]^ In order to determine the usefulness of these batteries, we lit a light‐emitting diode (LED) pattern with three batteries (Figure S10, Supporting Information). The experimental results showed a noticeable discrepancy from the theoretical capacity. The actual specific capacities of these two polymers were 85.7% and 51.4% of their theoretical capacities, which did not meet prior expectations. In comparison to the previously reported organic or polymer cathode materials, the active site utilization of TABQ‐DHBQ polymer was at a high level, as shown in Table S1 (Supporting Information). Consequently, its specific capacity was significantly higher than that of TAPT‐DHBQ polymer. However, the active site utilization of TABQ‐DHBQ polymer ultimately failed to exceed 90%, primarily due to the aggregation of linear polymers, which resulted in masking some active sites. To explain this discrepancy, we conducted density functional theory (DFT) calculations, as shown in Figure 3d,e. The selected computational units are depicted in Figure S11 (Supporting Information). As seen from electronic static potential (ESP) mappings (Figure 3d), the presence of the pyrazine group with high electronegative in TAPT‐DHBQ polymer attracted electrons from adjacent atoms, thus resulting in reduced electron cloud density on the neighboring carbonyl group. This would decrease the reactivity of the carbonyl group for Zn^2+^/H^+^ coordination, leading to the observed discrepancy between its actual and theoretical specific capacities. Additionally, the energy level simulations (Figure 3e) showed that the band gaps of the polymers were similar (1.42 and 1.46 eV), indicating comparable electrical conductivity.^[^ ^32^, ^59^ ^]^ However, due to the introduction of electron‐withdrawing group (pyrazine), the lowest unoccupied molecular orbital (LUMO) energy level of TAPT‐DHBQ polymer (−4.15 eV) was lower than that of TABQ‐DHBQ polymer (−3.56 eV), suggesting that TAPT‐DHBQ had higher electron affinity and was more likely to undergo coordination reactions with Zn^2+^/H^+^.^[^ ^60^ ^]^ Based on these two factors, the energy storage performance of the polymers was similar. The result suggested that the chemical environment around the active sites displayed a crucial role in determining their redox reactivity. The ESP mappings (Figure 3d) clearly indicated that the active sites (carbonyl groups) in TABQ‐DHBQ polymer were surrounded by electron‐donor groups with pronounced electropositive regions, which led to a high density of negative electron clouds around the active sites. As a result, the active sites in TABQ‐DHBQ polymer exhibited high redox reactivity for coordination with Zn^2+^/H^+^. In contrast, a highly electronegative pyrazine group was located near the active sites, competing for electrons and resulting in a dispersed negative electron cloud around the active sites. This reduced redox reactivity toward coordination with Zn^2+^/H^+^ contributed to the low utilization of the active sites. This finding suggested that, in the design of organic cathodes, a higher density of active sites did not necessarily translate into better performance. When designing high‐performance organic polymer cathodes of AZIBs, more attention should be also given to the electrochemical environment surrounding the active sites, rather than merely increasing the number of active sites.

The rate performance was further investigated by analyzing charge/discharge curves at various current densities (Figure 3f; Figure S12, Supporting Information). The charge/discharge curves of AZIBs based on TABQ‐DHBQ cathode exhibited excellent symmetry, with specific capacities of 325, 262, 214, 176, 135, and 90 mAh g^−1^ at the current density from 0.1 to 5 A g^−1^, respectively, and the recovered specific capacity was 283 mAh g^−1^ (87%). Meanwhile, TAPT‐DHBQ cathode exhibited the specific capacity of 280, 204, 169, 140, 111, and 70 mAh g^−1^ at the current density from 0.1 to 5 A g^−1^, respectively, and the recovered specific capacity was 218 mAh g^−1^ (78%). The results suggested that both polymers possessed large and continuous π‐conjugated structures.^[^ ^32^ ^]^ However, their recovered specific capacities were slightly lower, which will be further discussed in the electrochemical kinetics section. Additionally, as shown in Figure 3f, the specific capacities of these two polymers gradually approached each other at high charge/discharge current densities, and this phenomenon will also be further discussed in the electrochemical kinetics section. Furthermore, the long‐term cycling stability of these two polymer cathodes was evaluated at the current density of 5 A g^−1^. The results indicated that the specific capacities retention remained ≈90% and 95% of the initial specific capacity for TABQ‐DHBQ polymer and TAPT‐DHBQ polymer respectively after 2000 charge/discharge cycles, with the coulombic efficiency approaching 100% (Figure 3g). Interestingly, during the long‐term charge/discharge process, both polymers experienced a prolonged activation process. This phenomenon was primarily attributed to the acceleration of electron migration at high charge/discharge current densities, which led the electrode reaction to be increasingly dominated by surface capacitance. As a result, charge storage occurred through rapid surface adsorption/desorption, while the bulk embedding/desorption process of Zn^2+^/H^+^ was suppressed. This kinetic characteristic resulted in a significant activation process for both polymers.^[^ ^61^ ^]^ The long‐term cycling stability was further confirmed by UV–vis spectra, FT‐IR, SEM, and EDX. As shown in Figure S13 (Supporting Information), the UV–vis spectra of the aqueous electrolyte immersed electrode after 2000 charge/discharge cycles matched those of bare aqueous electrolyte. The FTIR spectra shown in Figure S14 (Supporting Information) demonstrated that the vibration peak positions and intensities of the characteristic C═O functional group did not significantly weaken or shift after 2000 charge/discharge cycles, confirming that the molecular structure of both polymers remained intact during the long‐term cycling process. The SEM images (Figure S15, Supporting Information) also revealed that the microscopic morphology of the two cathodes in the three different states remained highly consistent, including particle sizes and surface roughness. No pulverization, or agglomeration was observed, indicating excellent mechanical tolerance due to the flexible skeleton of organic polymers and the 2D layer‐by‐layer stacking structures with larger interlayer spacing. The EDX images (Figures S16 and S17, Supporting Information) further showed that the elements were evenly distributed before and after the long‐term cycling process, without any local enrichment or phase separation, suggesting that reversible coordination reaction and adsorption occurred. Additionally, XRD, SEM images, and EDX analysis of the zinc anode were also performed in their initial state, after 1000 charge/discharge cycles, and after 2000 charge/discharge cycles to investigate the stability of the anode. XRD patterns (Figure S18, Supporting Information) indicate minimal to no formation of side products on the Zn electrode following cycling. SEM observations (Figure S19, Supporting Information) corroborate this finding, displaying no significant dendrite formation or morphological degradation of the Zn anode surface after prolonged cycling. Furthermore, EDX mappings (Figures S20 and S21, Supporting Information) confirm negligible presence of sulfur‐based side‐products, reflecting minimal parasitic reactions.

**Figure 4:**
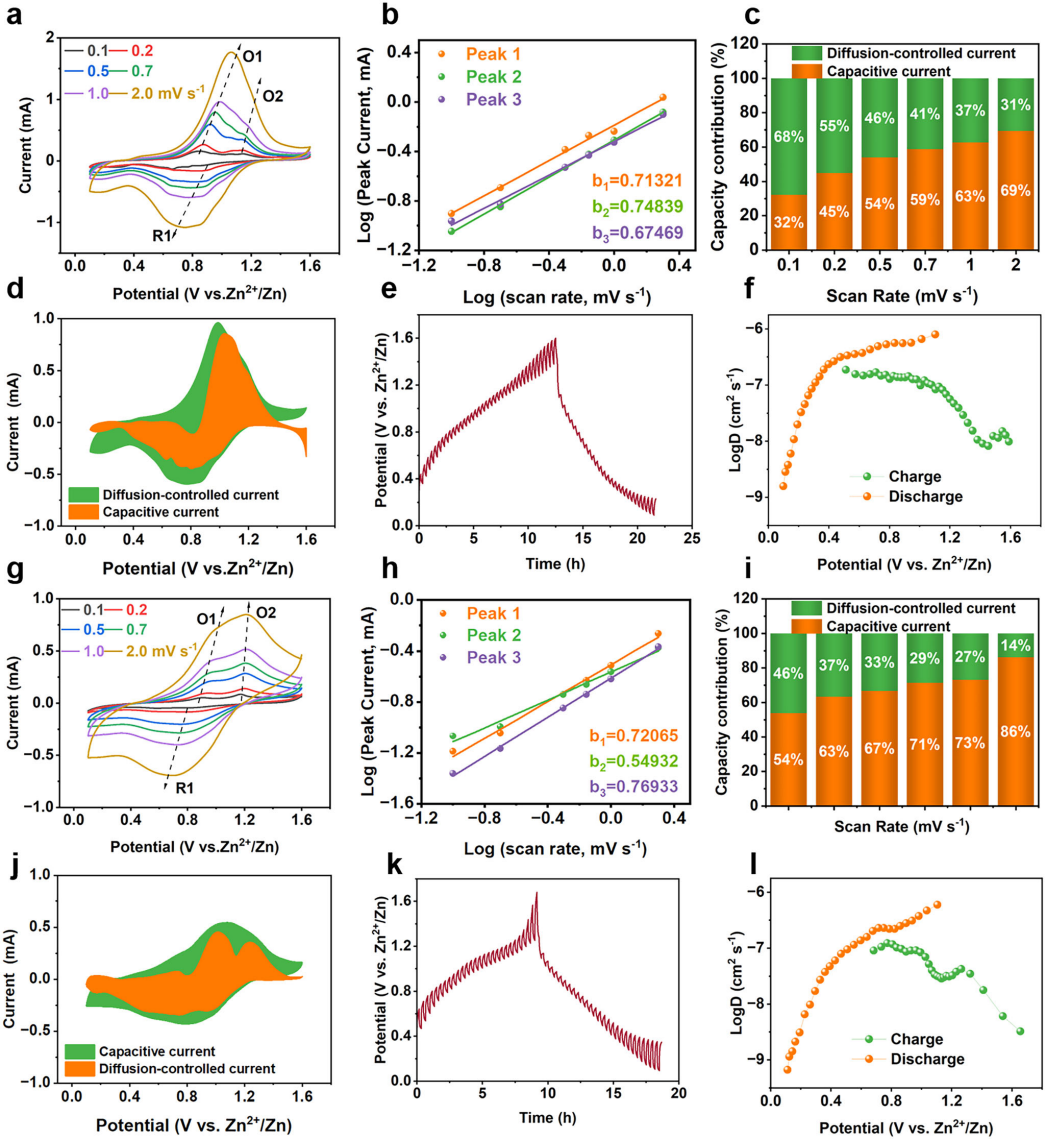
The kinetics investigation of TABQ‐DHBQ and TAPT‐DHBQ cathodes. a, g) CV curves of TABQ‐DHBQ (a) and TAPT‐DHBQ (g) cathodes at different scan rates from 0.1 to 2.0 mV s^−1^. b, h) Linear relationship of TABQ‐DHBQ (b) and TAPT‐DHBQ (h) cathodes between redox current and scan rate for fitting *b*‐values. c, i) Capacitive contribution of TABQ‐DHBQ (c) and TAPT‐DHBQ (i) cathodes at the scan rate of 1 mV s^−1^. d, j) Capacitive and diffusion‐controlled charge storage relative contributions of TABQ‐DHBQ (d) and TAPT‐DHBQ (j) cathodes at different scan rates. e, k) GITT curves of TABQ‐DHBQ (e) and TAPT‐DHBQ (k) cathodes. f, l) *D* value estimated from GITT curves of TABQ‐DHBQ (f) and TAPT‐DHBQ (l) cathodes.

The electrochemical kinetics of the polymers were evaluated by measuring CV curves at different scan rates, ranging from 0.1 to 2 mV s^−1^ (**Figure** **4**). Notably, two distinct pairs of redox peaks were clearly observed in the CV curve of TABQ‐DHBQ at low scan rates, with the oxidation peaks appearing at 0.85, 1.13 V, and the reduction peaks at 0.74 V (Figure 4a). Minimal variation in peak potentials was observed at different scan rates, suggesting a stable and reversible kinetic process during the charge/discharge process.^[^ ^61^ ^]^ However, as the scan rate increased, one pair of redox peaks gradually became less prominent, indicating that the rapid response of the active sites at high scan rates was unsatisfactory. As shown in Figure 4b, further analysis of the relationship between scan rate (*v*) and redox peaks current (*i*) revealed *b*‐values close to 0.5 (ranging from 0.6 to 0.7), demonstrating that the charge/discharge process was primarily controlled by diffusion.^[^ ^62^ ^]^ The contributions of diffusion‐controlled and capacitance‐controlled processes were further analyzed, as shown in Figure 4c,d. It was observed that the diffusion‐controlled contribution dominated at low scan rates. As the scan rate increased, the contribution of capacitive control gradually increased, which would reach a maximum of only 63% (Figure 4c). The electrochemical kinetics results could explain well the unsatisfactory rate performance and long‐term cycling stability of TABQ‐DHBQ polymer. In contrast, the electrochemical kinetics investigation of TAPT‐DHBQ revealed that its kinetics were controlled by both capacitance and diffusion (Figure 4g–j). Compared with TABQ‐DHBQ polymer, TAPT‐DHBQ polymer exhibited a higher interlayer spacing, which enhanced the proportion of capacitive‐controlled. This difference resulted in a significant disparity in the specific capacities of the two polymers at low charge/discharge current densities, while their specific capacities gradually approached each other as the charge/discharge current densities increased. At low charge/discharge current densities, the energy storage process of AZIBs was primarily controlled by diffusion, with specific capacities depending on the reversible coordination of Zn^2+^/H^+^ in the polymers.^[^ ^63^ ^]^ The TABQ‐DHBQ polymer, with more ordered layer‐by‐layer stacking, exhibited a higher diffusion‐dominated specific capacity of 68% at low charge/discharge current densities, compared to TAPT‐DHBQ polymers (46%). At high charge/discharge current densities, the energy storage kinetics gradually shifted toward surface capacitance behavior.^[^ ^64^ ^]^ In this case, charge storage was governed by rapid ions adsorption/desorption at the electrode/electrolyte interfaces, weakening the impact of structural differences, which resulted in the specific capacities of the two polymers becoming similar at high charge/discharge current densities. Motivated by these findings, galvanostatic intermittent titration technique (GITT) tests were conducted on TABQ‐DHBQ cathode to probe ion‐diffusion kinetics, as shown in Figure 4e,f. The diffusion coefficient (*D*) decreased gradually from 10^−6^ to 10^−8^ cm^2^ s^−1^ during the discharge process. Similarly, the GITT tests were performed on TAPT‐DHBQ cathode, yielding a *D* value that decreased from 10^−7^ to 10^−8^ cm^2^ s^−1^ during the discharge process (Figure 4k.l). The high *D* values of the two polymers were primarily attributed to their 2D structures, and the difference in *D* values of the two polymers further indicated the difference in energy storage kinetics between them, mainly corresponded to the highly ordered layer‐by‐layer stacking structure of TABQ‐DHBQ polymer and the wide interlayer spacing of TAPT‐DHBQ polymer.^[^ ^65^ ^]^

**Figure 5:**
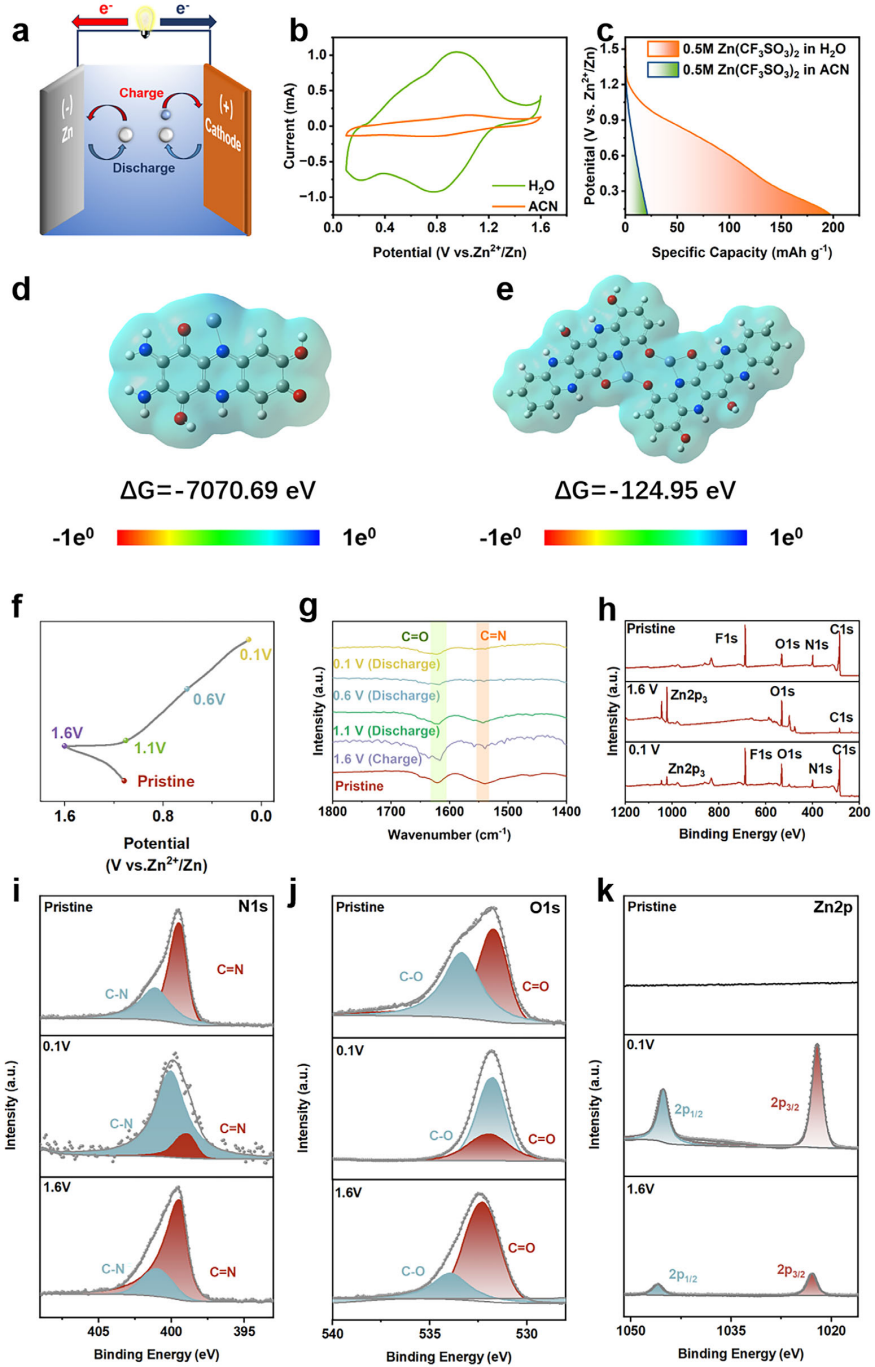
The energy storage mechanism investigation of TABQ‐DHBQ and TAPT‐DHBQ cathode. a) Schematic diagram of energy storage mechanisms for TABQ‐DHBQ and TAPT‐DHBQ cathodes. b, c) CV curves (b) at the scan rate of 1 mV s^−1^ and GCD (c) curves at the current density of 0.1 A g^−1^ of TABQ‐DHBQ cathode in 0.5 M Zn(CF_3_SO_3_)_2_ electrolyte with deionized water or anhydrous acetonitrile as solvent respectively. d, e) The mostly possible discharge configurations of TABQ‐DHBQ polymer (d) and TAPT‐DHBQ polymer (e) with their binding energy. f, g) Ex‐situ FTIR spectra of TABQ‐DHBQ cathode at different charge/discharge states. h) Ex‐XPS spectrum of TABQ‐DHBQ cathode at different charge/discharge states. i–k) High‐resolution XPS spectra of N1s (g), O1s (h), and Zn2p (i) of TABQ‐DHBQ cathode at different charge/discharge states.

Finally, we investigated the charge storage mechanisms of these two polymers through electrochemical measurements, ex‐situ FT‐IR and ex‐situ XPS analyses (**Figure** **5**). Initially, we examined the types of inserted cations by utilizing a 0.5 M Zn(CF_3_SO_3_)_2_ electrolyte, with either deionized water or anhydrous acetonitrile as the solvent. As shown in Figure 5b,c, the specific capacity of TABQ‐DHBQ cathode was significantly higher in the aqueous electrolyte (197 mAh g^−1^) compared to the anhydrous acetonitrile electrolyte (20 mAh g^−1^). The same results are shown in Figure S22 (Supporting Information), the specific capacity of TAPT‐DHBQ cathode was 202 mAh g^−1^ in aqueous electrolyte, while the specific capacity was 54 mAh g^−1^ in anhydrous acetonitrile electrolyte. The results suggested that the energy storage mechanisms of TABQ‐DHBQ and TAPT‐DHBQ cathodes were mainly based on the insertion of H^+^. Subsequent calculations revealed that the contribution of H^+^ insertion to the total capacity was ≈90% for TABQ‐DHBQ cathodes and 76% for TAPT‐DHBQ cathodes. The TABQ‐DHBQ polymer exhibited a significantly higher proportion of H^+^ energy storage. The active sites for Zn^2+^/H^+^ storage in both polymers were C═O and C═N.^[^ ^66^ ^]^ DFT calculations (Figure S23b,i, Supporting Information) showed that the binding Gibbs free energy (Δ*G*) of the active sites in TABQ‐DHBQ polymer with H^+^ was −78.58 eV, which was significantly lower than the Δ*G* of the active sites in TAPT‐DHBQ polymer with H^+^ (−27.13 eV), indicating that H^+^ preferentially occupied the active sites within TABQ‐DHBQ polymer. This was mainly because the benzoquinone group in TABQ‐DHBQ polymer exhibited a strong electron‐withdrawing effect, which reduced the electron density of the active oxygen atoms and enhanced their ability to bind with H^+^, whereas the conjugated effect of the pyrazine ring in TAPT‐DHBQ polymers dispersed the charge and weaken the specificity of these active sites for H^+^. Therefore, TABQ‐DHBQ polymer exhibited a higher H^+^ storage ratio. Additionally, DFT calculations further optimized the possible structures of the two polymers coordinating with Zn^2+^/H^+^, as shown in Figure 5d,e and Figure S23 (Supporting Information). One TABQ‐DHBQ polymer coordinating with one Zn^2+^ and one H^+^ through two C═O and one C═N active sites exhibited the lowest Δ*G* of −7070.69 eV, indicating the most likely discharge configuration (Figure 5d). In contrast, the most likely discharge configuration of TAPT‐DHBQ polymer (Figure 5e) was to bind eight H^+^ and two Zn^2+^ with eight C═O and six C═N active sites by two polymer molecules (Δ*G *═ −124.95 eV). Therefore, the energy storage contributions of C═O and C═N in TABQ‐DHBQ polymer were 66.7% and 33.3% respectively, while the energy storage contributions of C═O and C═N in TAPT‐DHBQ polymer were 57.1% and 42.9% respectively. Next, the ex‐situ FTIR spectra were employed to further elucidate the charge storage mechanism by examining the molecular structures in the pristine state, fully discharged state (0.1 V), fully charged state (1.6 V), and discharged state (1.1 and 0.6 V) (Figure 5f,g). Clearly, the characteristic peak of the C═O group at 1620 cm^−1^ was significantly weaker at the fully discharged state (0.1 V) compared to the pristine state, indicating the successful occurrence of H^+^ and Zn^2+^ coordination reaction. The intensity of this characteristic peak gradually returned to its original state after charging to 1.6 V, demonstrating the reversibility of H^+^ and Zn^2+^ coordination reaction for energy storage via the C═O group. The same trend was observed on the C═N characteristic peak at 1545 cm^−1^, which suggested that the C═N group was also involved in the reversible H^+^ and Zn^2+^ coordination reaction. Lastly, the XPS spectra corroborated these results, as shown in Figure 5h–k. In the high‐resolution O1s XPS spectra, the intensity of the C═O characteristic peak was diminished, while that of the C‐O characteristic peak was enhanced after full discharge to 0.1 V, and both returned to their initial intensities after full charge to 1.6 V. The same trend was observed on the N1s XPS spectra, which suggested that the C═N group was also involved in the reversible H^+^ and Zn^2+^ coordination reaction. Additionally, the high‐resolution Zn2p XPS spectra exhibited similar trends, with characteristic peaks emerging after full discharge to 0.1 V and diminishing in intensity after full charge to 1.6 V, indicating the presence of residual Zn^2+^ during the charge/discharge process. For comparison, the TAPT‐DHBQ cathode exhibited a similar energy storage mechanism (Figures S24 and S25, Supporting Information). The results suggested that the energy storage mechanism of both polymers involved Zn^2+^/H^+^ coordination reactions.

**Figure 6:**
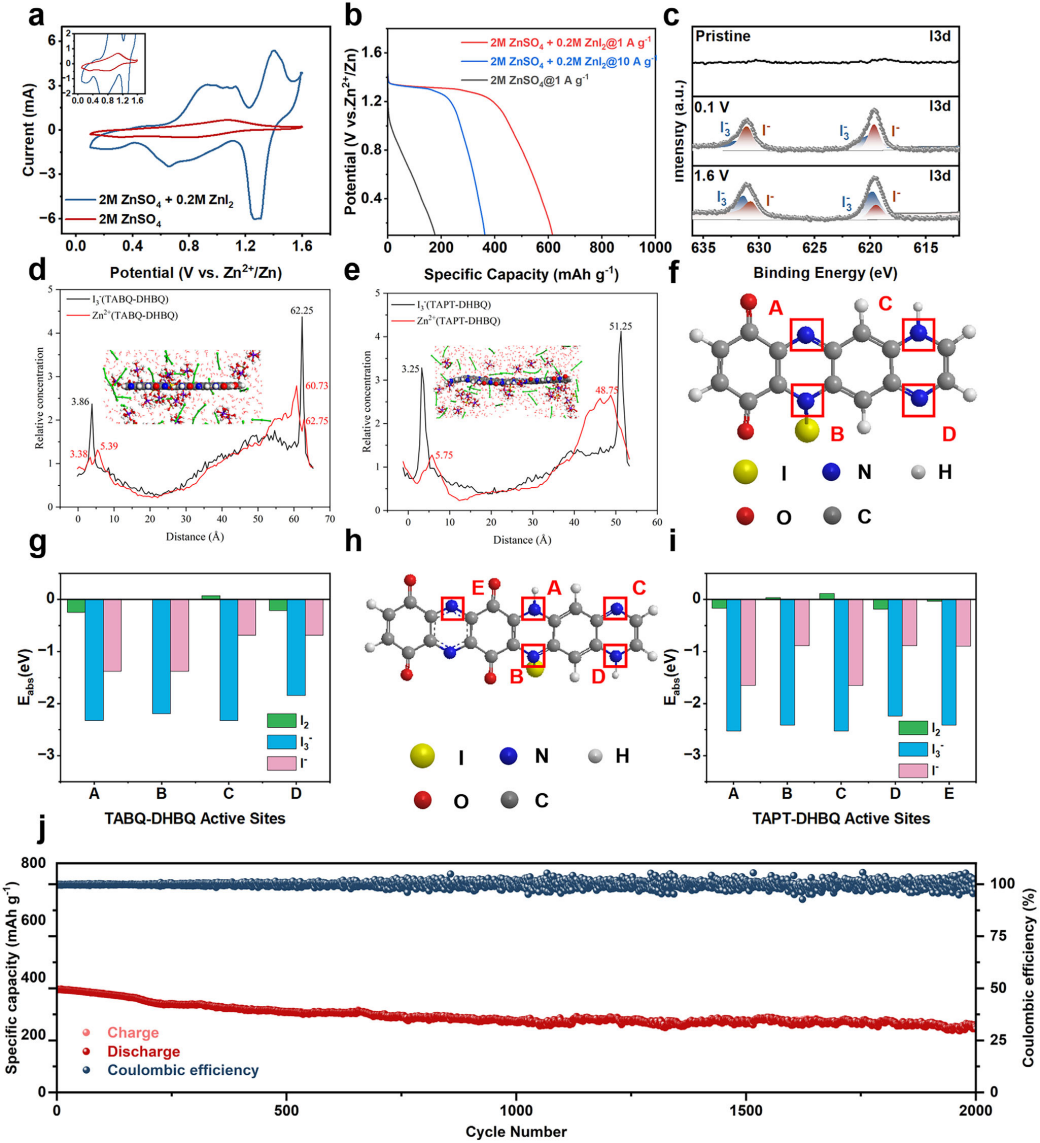
a) The CV curves of Zn//TABQ‐DHBQ batteries with or without ZnI_2_ electrolyte additive respectively at the scan rate of 1 mV s^−1^. b) The GCD curves of Zn//TABQ‐DHBQ batteries with or without ZnI_2_ electrolyte additive respectively at the current density of 1 A g^−1^. c) High‐resolution XPS spectra of I3d of TABQ‐DHBQ cathode at different charge/discharge states. d, e) The relative concentration of Zn^2+^ and I_3_^−^ ions curves for TABQ‐DHBQ polymer (d) and TAPT‐DHBQ polymer (e). f–i) The DFT calculations for the adsorption process between iodine, iodide ions, triiodide ions and the TABQ‐DHBQ polymer (f, g) or TAPT‐DHBQ polymer (h, i). j) Long‐term cycling stability of Zn||TABQ‐DHBQ batteries with ZnI_2_ electrolyte additive carried out at the current density of 10 A g^−1^.

To further enhance the commercialization potential of these AZIBs, iodide was incorporated as an active redox additive into the electrolyte, aiming to improve the energy storage performance of Zn//TABQ‐DHBQ batteries under high current density charge/discharge conditions. Based on cost considerations, ZnI_2_, KI, NH_4_I, and tetraethylammonium iodide (TEA‐I), which were relatively inexpensive, were selected as additives to test the energy storage performance of AZIBs and to make a horizontal comparison. As shown in Figure S26 (Supporting Information), the performance of the AZIBs‐based two polymer cathodes with ZnI_2_ additive was significantly better than those of the other iodide additives, with a specific capacity of 618 mAh g^−1^ (1 A g^−1^) for Zn||TABQ‐DHBQ battery and a specific capacity of 607 mAh g^−1^ (1 A g^−1^) for Zn||TAPT‐DHBQ battery, respectively. This was primarily due to the influence of different cations. K^+^ competed with the reversible coordination of Zn^2+^, and the XRD patterns show the K^+^ formed an inactive alloy phase (KZn_13_) with Zn^2+^ (as shown in Figure S27, Supporting Information), thereby reducing the concentration of active substances in the AZIBs system. NH_4_^+^ produced NH_3_ through the reduction process on the anode surface, which affected the stability of the AZIBs system. The large volume of TEA organic cations resulted in significantly higher R_ct_ compared to the ZnI_2_ additive‐based AZIBs (Figure S28, Supporting Information), leading to a marked reduction in performance. Consequently, this study ultimately selected ZnI_2_ as the electrolyte additive. On this basis, further investigation was conducted into the effect of different concentrations of ZnI_2_ additives on AZIBs performance (Figure S29, Supporting Information). In Zn||TABQ‐DHBQ batteries, the results indicated that the specific capacities did not increase accordingly with the ZnI_2_ concentration increased after that exceeding 0.2 m. In Zn||TAPT‐DHBQ batteries, the 0.2 m ZnI_2_ additive system exhibited the highest specific capacity. When the concentration was lower than 0.2 m, the iodide concentration was insufficient, limiting its promotion of the Zn^2+^ redox process at the cathode surface. When the concentrations exceeded 0.2 m, the high iodide concentration could trigger the shuttle effect of polyiodides, where soluble polyiodides diffused in the electrolyte and adsorbed to the cathode. This produced a competitive mechanism with the coordination of Zn^2+^, leading to a decrease in specific capacity. Therefore, 0.2 m ZnI_2_ was ultimately determined to be the optimal electrolyte additive. Compared to the ZnSO_4_ aqueous electrolyte, the CV curves of Zn||TABQ‐DHBQ batteries (**Figure** **6**a) revealed significantly enlarged redox peaks for the storage of Zn^2+^/H^+^, along with a substantial increase in the integral area of the CV curves upon the addition of ZnI_2_ electrolyte additive, indicating enhanced energy storage performance. This improvement was primarily attributed to the adsorption of iodides on the surface of the organic cathodes, which reduced the R_ct_ and ohmic resistance (R_s_) at the electrode/electrolyte interface. The equivalent circuits inset of Figure S30 (Supporting Information) were derived by fitting Nyquist curves of ZnSO_4_ electrolyte and ZnSO_4_ + ZnI_2_ electrolyte in Zn||TABQ‐DHBQ batteries respectively. The fitting results revealed that R_s_ decreased from 6.4 to 2.0 Ω, while R_ct_ decreased from 32.9 to 10.8 Ω upon the addition of ZnI_2_ electrolyte additive, promoting the reversible coordination reaction between Zn^2+^/H^+^.^[^ ^6^ ^]^ The same phenomenon also occurred in Zn||TAPT‐DHBQ batteries, where the CV integral area also increased significantly (Figure S31a, Supporting Information), R_ct_ decreased from 34.0 to 11.2 Ω, and R_s_ decreased from 8.2 to 2.5 Ω (Figure S32, Supporting Information). This reduction in resistance was a key factor contributing to the improved energy storage performance of AZIBs with ZnI_2_ electrolyte additive. In addition, after iodides were adsorbed on the surface of the two polymers, the electrical conductivities of the cathodes were significantly enhanced, as confirmed by the I‐V curves (Figure S33, Supporting Information). The improved electrical conductivity facilitated accelerated electron transfer, thereby supporting the AZIBs to charge/discharge rapidly at high current densities. Therefore, the specific capacity of Zn||TABQ‐DHBQ battery increased significantly from 176 to 618 mAh g^−1^ at a high current density of 1 A g⁻¹ after the addition of ZnI_2_ electrolyte additive, while the specific capacity of 360 mAh g^−1^ was achieved at a high current density of 10 A g^−1^ (Figure 6b). And the specific capacity of Zn||TAPT‐DHBQ battery increased significantly from 140 to 607 mAh g^−1^ at a high current density of 1 A g⁻¹ after the addition of ZnI_2_ electrolyte additive, while the specific capacity of 354 mAh g^−1^ was achieved at a high current density of 10 A g^−1^ (Figure S31b, Supporting Information). These results demonstrated superb rate performance, which was of significant importance for its commercial application. This increase in electrical conductivity was another key factor contributing to the improved energy storage performance of AZIBs with ZnI_2_ electrolyte additive. Additionally, the appearance of a new pair of redox peaks (1.41 and 1.24 V) in CV curves confirmed the participation of a novel redox pair (I^−^/I_3_^−^).^[^ ^67^ ^]^ Notably, the discharge plateau became more pronounced, and the discharge voltage significantly improved to 1.28 V following the involvement of ZnI_2_ electrolyte additive. Consequently, the energy density of Zn||TABQ‐DHBQ battery increased significantly from 206 to 678.62 Wh kg^−1^ upon the addition of ZnI_2_ electrolyte additive. Zn||TAPT‐DHBQ battery also exhibited a high energy density of 670.7 Wh kg^−1^. This result was further corroborated by ex‐situ XPS characterization (Figure 6c). In the high‐resolution I3d XPS spectra, the intensity of the I₃⁻ characteristic peak at 619.7 and 631.5 eV significantly decreased after full discharge to 0.1 V, whereas the intensity of the I⁻ characteristic peak at 619.0 and 630.8 eV increased. Conversely, upon full charge to 1.6 V, the intensity of the I⁻ characteristic peak decreased, while the intensity of the I₃⁻ characteristic peak increased, indicating the conversion of I⁻ to I₃⁻.^[^ ^68^ ^]^ To further investigate the action mechanism of ZnI_2_ additive, we examined the performance of AZIBs using pure ZnI_2_ electrolyte and ZnSO_4_‐ZnI_2_ mixed electrolyte (Figure S34, Supporting Information). The results showed that the specific capacity of the two polymers in pure ZnI_2_ electrolyte was lower than that in the ZnSO_4_‐ZnI_2_ mixed electrolyte, with values of 339 mAh g^−1^ (TABQ‐DHBQ) and 318 mAh g^−1^ (TAPT‐DHBQ) at 10 A g^−1^, respectively. These findings indicated that ZnSO_4_ remained a critical source of Zn^2+^ in the electrolyte. When pure ZnI_2_ was used as the electrolyte, its concentration was limited by the shuttle effect of polyiodide species, preventing it from providing enough Zn^2+^ for energy storage. Additionally, the shuttle effect of polyiodide species led to a significant loss of active materials in the cathodes and contributed to self‐discharge.^[^ ^69^ ^]^ Furthermore, molecular dynamics (MD) simulations were conducted to investigate the adsorption of triiodine ions (I_3_^−^) and hydrated zinc ions ([Zn(H_2_O)_6_]^2+^) on the surface of the two polymers (Movies S1 and S2, Supplementary Movie). Based on this simulation data, the equilibrium adsorption configurations of these two polymers with I_3_^−^ and [Zn(H_2_O)_6_]^2+^ ions were optimized, and radial density distribution curves were determined (Figure 6d,e). The results revealed that both I_3_^−^ and [Zn(H_2_O)_6_]^2+^ ions were strongly adsorbed on the surfaces of the two polymers. However, the [Zn(H_2_O)_6_]^2+^ ions were closer to the polymer surface and exhibited higher density than I_3_^−^, indicating that the binding of [Zn(H_2_O)_6_]^2+^ to the polymers was stronger and more easily adsorbed, suggesting that Zn^2+^ remained the primary ions for energy storage. The enhanced energy storage performance at high charge/discharge current density after adding ZnI_2_ electrolyte additive was primarily attributed to iodine adsorption on the surface of TABQ‐DHBQ cathode during the charging process, promoting the revisable coordination of Zn^2+^.^[^ ^6^, ^70^, ^71^, ^72^ ^]^ Furthermore, DFT calculations were used to optimize the adsorption mechanism between iodine, iodide ions, triiodine ions, and the two polymers, as shown in Figure 6f–i. The N sites in both polymers acted as the active adsorption sites for iodine, iodide ions, and triiodine ions (Figure 6f,h). The difference in adsorption energy barriers between iodide ions, triiodine ions, and iodine and the difference active sites in two polymers indicated that the adsorption active sites of TABQ‐DHBQ polymer were A and B sites, while the adsorption active sites of TAPT‐DHBQ polymer were A and C sites (Figure 6g,i). In comparison, the adsorption energy barrier of triiodine ions was lower than that of iodide ions and iodine, which allowed for better fixation of triiodine ions and reduced their shuttle effect, thereby improving the stability of the AZIBs. Therefore, as shown in Figure 6j, after 2000 charge/discharge cycles at high current density (10 A g^−1^), the specific capacity of Zn||TABQ‐DHBQ battery still maintained 63.2%, with only 0.067 mAh g^−1^ decline per cycle. Meantime, the coulombic efficiency remained ≈100%. Zn||TAPT‐DHBQ battery also displayed a similar long‐term cycling stability (Figure S31c, Supporting Information). To investigate the effect of ZnI_2_ additive on the cathode during long‐term charge/discharge cycles, FT‐IR, SEM, and EDX analyses were performed on the cathode structure before and after 2000 charge/discharge cycles. The FT‐IR spectra as shown in Figure S35 (Supporting Information) indicated that the position and intensity of the characteristic peaks (C═O, C═N) did not shift or weaken significantly after 2000 charge/discharge cycles, suggesting that the introduction of ZnI_2_ did not induce irreversible structural changes in the two polymers. The SEM images (Figure S36, Supporting Information) further confirmed that the surface morphology of the cathodes after 2000 charge/discharge cycles remained highly consistent with its initial state, with no powdering or agglomeration, demonstrating that the ZnI_2_ additive did not disrupt the microstructural integrity of the cathodes. This is primarily attributed to the wide interlayer spacing in the 2D layer‐by‐layer stacking structure of the two polymers. Additionally, the EDX images (Figures S37 and S38, Supporting Information) showed that the Zn and I elements were evenly distributed on the electrode surface, indicating that iodide ions were adsorbed onto the electrode surface during the charge/discharge process, which reduced the R_ct_ at the electrode/electrolyte interface and promoted the rapid coordination of Zn^2+^. These results demonstrated that the introduction of ZnI_2_ had no negative impact on the structural stability of the cathode materials. Similarly, the anode exhibited excellent structural stability, as evidenced by the XRD patterns, which showed almost no by‐products (Zn_4_SO_4_(OH)_6_) on the Zn anode after 2000 charge/discharge cycles (Figure S39, Supporting Information). This behavior was primarily attributed to the strong adsorption affinity of the two polymers for triiodine ions, effectively immobilizing polyiodide species and mitigating their shuttle effect.

## 3 Conclusion

In summary, we designed and prepared two 2D linear polymers (namely TABQ‐DHBQ and TAPT‐DHBQ) to investigate the relationship between energy storage active site density and their Zn^+^ energy storage performance. The experimental results indicated that a higher density of active sites did not necessarily lead to better performance in the design of organic cathodes. Specifically, the actual specific capacities of these two polymers were 325 mAh g^−1^ (TABQ‐DHBQ) and 280 mAh g^−1^ (TAPT‐DHBQ), corresponding to 85.7% and 51.4% of their theoretical capacities respectively. DFT calculations further demonstrated that an excessively high density of active sites led to competitive interactions between neighboring active sites, thereby reducing their effective utilization. Consequently, despite TAPT‐DHBQ polymer having a higher number of active sites, its actual specific capacity was only comparable to that of TABQ‐DHBQ polymer. When designing high‐performance organic polymer cathodes of AZIBs, greater emphasis should be placed on the electrochemical environment surrounding the active sites, rather than solely focusing on increasing the number of active sites. By adding the ZnI_2_ electrolyte additive, iodide ions significantly promoted the Zn^2+^/H^+^ revisable coordination. As a result, the energy storage performance of TABQ‐DHBQ cathode was further improved to 618 mAh g^−1^ at a current density of 1 A g^−1^, with an energy density of 678.62 Wh kg^−1^, while the specific capacity of TAPT‐DHBQ cathode has been enhanced to 607 mAh g^−1^ at a current density of 1 A g^−1^ with an energy density of 670.7 Wh kg^−1^. This finding offers new insights for the design of polymer cathode materials for efficient AZIBs in future applications.

## Conflict of Interest

The authors declare no conflict of interest.

## Supporting information



Supporting Information

Supplementary MovieS1

Supplementary MovieS2

## Data Availability

Research data are not shared.
