# Supplementary material for: Linear Polymer Cathode Materials for Highly Efficient Aqueous Zinc‐Ion Batteries: Is the High Active Sites Density Necessary?
Source: Adv Sci (Weinh). 2025 Apr 26;12(27):2503156. doi: 10.1002/advs.202503156 (PMC12279229; doi:10.1002/advs.202503156)
Supplement: Supplementary file 1 — Supporting Information [file ADVS-12-2503156-s002.docx]

Supplementary Information

**Linear Polymer Cathode Materials for Highly Efficient Aqueous Zinc-Ion Batteries: Is the High Active Sites Density Necessary?**

*Yiyang Dai^1^, Yao Yao^1, *^, Liang Feng^1^, Zhenglong Qiu^1^, Min Deng^1^, and Qiang Peng^1,2,*^*

^1^College of Materials and Chemistry & Chemical Engineering, Chengdu University of Technology, Chengdu 610059, P. R. China

^2^School of Chemical Engineering and State Key Laboratory of Polymer Materials Engineering, Sichuan University, Chengdu 610065, P. R. China.

^*^Corresponding authors: [yyao1994@cdut.edu.cn](mailto:yyao1994@cdut.edu.cn); [qiangpeng@scu.edu.cn](mailto:qiangpeng@scu.edu.cn)

**Experimental Section**

1. **Materials**

Methanol (MeOH), N,N-dimethylformamide (DMF), acetone, dry acetonitrile (CH_3_CN), N-methylpyrrolidone (NMP), phosphoric acid (H_3_PO_4_), hydrazine hydrate, sodium acetate, hydrochloric acid (HCl), 2,3,5,6-tetraaminocyclohexa-2,5-diene-1,4-dione (TABQ), 2,3,7,8-tetraaminophenazine-1,4,6,9-tetraone (TAPT), 2,5-dihydroxycyclohexa-2,5-diene-1,4-dione (DHBQ) were purchased from Adamas. Chloranil (TCBQ), potassium phthalimide were purchased from Shanghai Aladdin Biochemical Technology Co., Ltd. All reagents and solvents were used without further purification.

1. **Materials synthesis**
   1. **Synthesis of the 2,3,5,6-tetraaminocyclohexa-2,5-diene-1,4-dione (TABQ).**

As depicted in **Scheme S1a**, chloranil (4.92 g, 20.0 mmol) and potassium phthalimide (15.24 g, 82.3 mmol) were introduced into a round-bottom flask containing 80 mL of dry acetonitrile. The reaction mixture was stirred at reflux under an inert atmosphere for 18 hours, resulting in the formation of a dark brown, sludge-like solid. This solid was initially subjected to three washes with a 600 mL water/ethanol (1:1, v:v) solvent mixture. The dark purple solution was carefully decanted, yielding a brown solid, which was then further washed three times with 600 mL of boiling DMF. The product was isolated by vacuum filtration and subsequently washed with ethanol. After drying under reduced pressure, 9.10 g of yellow powder was obtained, corresponding to a yield of 68%. In a two-neck round-bottom flask, the resulting yellow powder was treated with hydrazine monohydrate (40 mL, 840 mmol), added dropwise under an inert atmosphere over the course of one hour at room temperature. The mixture was then stirred for an additional hour, followed by further stirring for two hours at 80°C. The reaction mixture was isolated *via* vacuum filtration and washed with water and ethanol, affording 1.98 g of lustrous purple crystalline solid with an 85% yield. Its structure was determined by ^1^H nuclear magnetic resonance (NMR) spectrum (**Scheme S1b**).


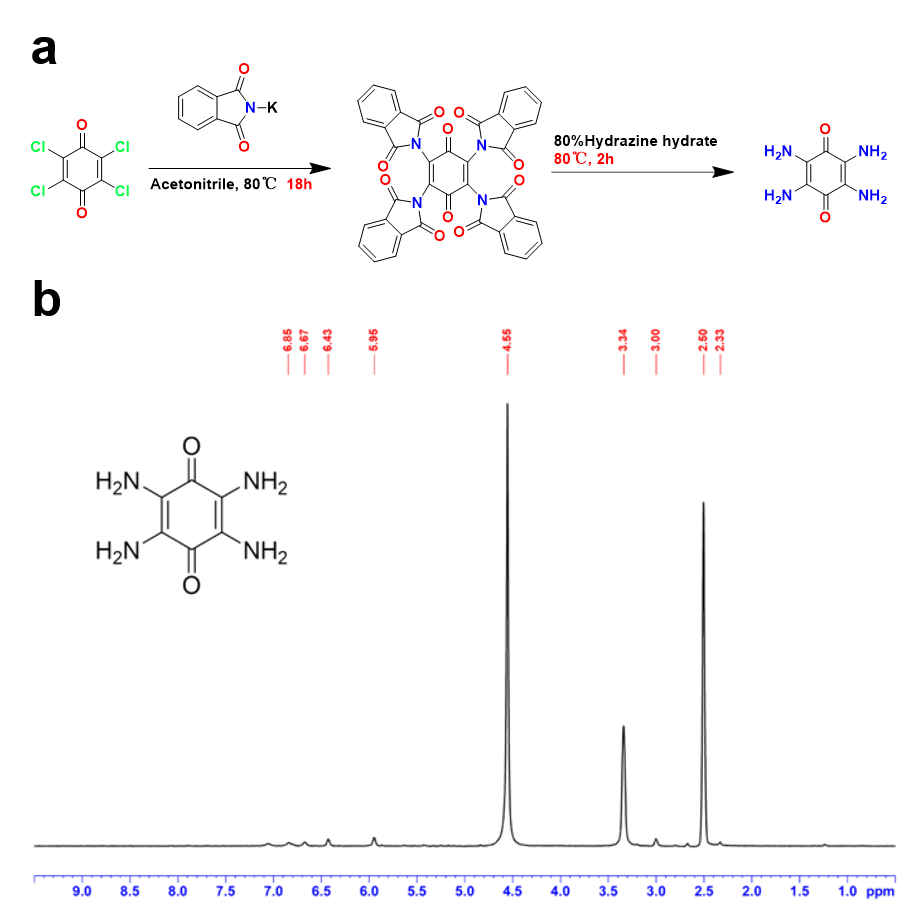


**Scheme S1.** (a) The synthesis route of TABQ. (b) ^1^H-NMR spectrum of TABQ.

- 1. **Synthesis of the 2,3,7,8-tetraaminophenazine-1,4,6,9-tetraone (TAPT).**

As illustrated in **Scheme S2a**, 1.68 g (10 mmol) of TABQ and 6.56 g (80 mmol) of sodium acetate were dispersed in 100 mL of deionized water, followed by the addition of 40 mmol of concentrated HCl. The mixture was sonicated for 15 minutes. Subsequently, the mixture was heated to reflux under an air stream for 5 hours at 85°C. After cooling to room temperature, the mixture was filtered and washed sequentially with water and acetone. The resulting TAPT was then dried under vacuum at 80°C overnight, yielding 86%. Its structure was determined by ^1^H NMR spectrum (**Scheme S2b**).


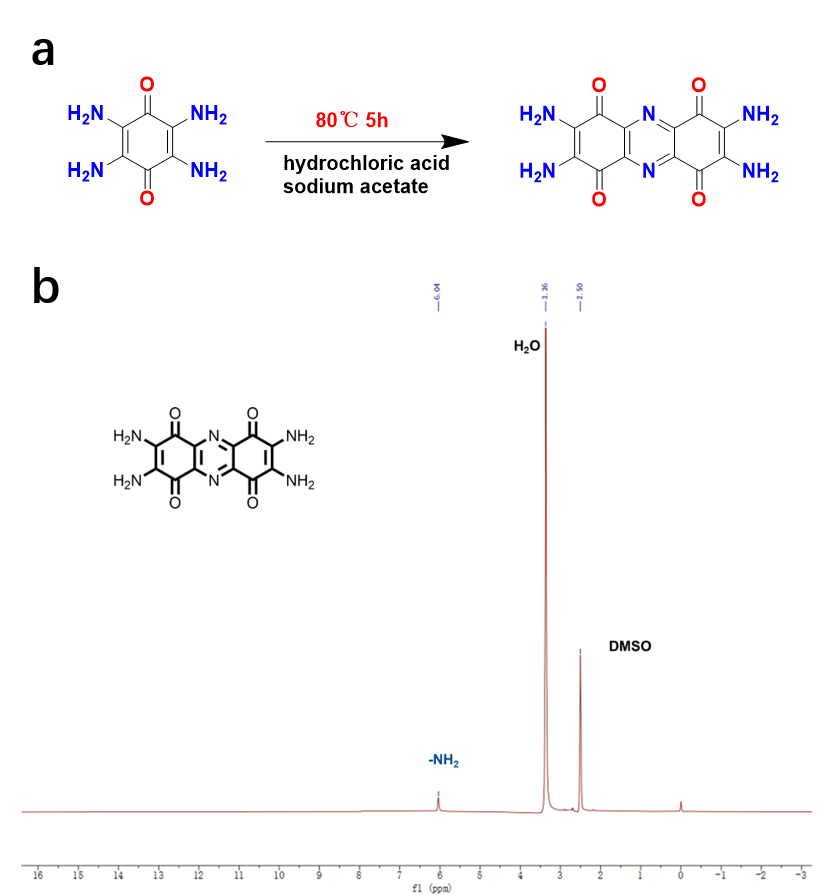


**Scheme S2.** (a) The synthesis route of TAPT. (b) ^1^H-NMR spectrum of TAPT.

- 1. **Synthesis of the TABQ-DHBQ and TAPT-DHBQ.**

As depicted in **Scheme S3**, the synthesis of these two polymeric materials was carried out according to previously reported procedures. TABQ (336 mg, 2 mmol) or TAPT (600 mg, 2 mmol) and DHBQ (280 mg, 2 mmol) were added to a round-bottom flask under a nitrogen atmosphere at room temperature. A solution of NMP (20 mL), containing a few drops of H₃PO₄ or HCl, was slowly introduced to dissolve the reactants. After stirring for 30 minutes, the mixture was heated to 100°C under nitrogen and maintained in an oil bath for 12 hours. Upon cooling to room temperature, the resulting black precipitate was collected by suction filtration. The crude product was washed successively with methanol, deionized water, and ethanol several times. The obtained black solid was then subjected to Soxhlet extraction using methanol, acetone, deionized water, and ethanol in sequence. Finally, the product was dried under vacuum at 60°C for 12 hours, yielding a black powder (277 mg or 352 mg).


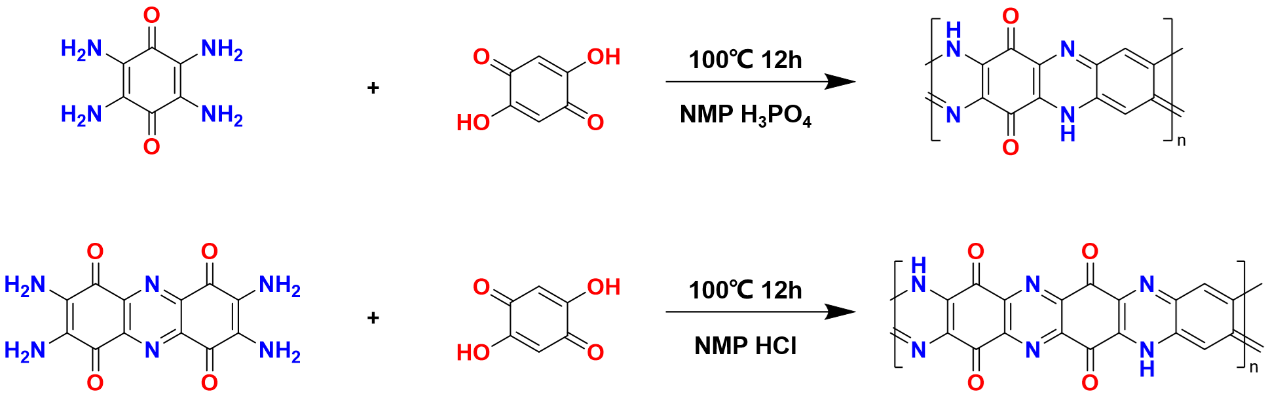


**Scheme S3.** The synthesis route of TABQ-DHBQ and TAPT-DHBQ polymers.

1. **Characterization**

The molecular weight analysis of the two polymers were performed by gel permeation chromatography (GPC, 1260 infinity II, Agilent, the United Kingdom) at 150 ^o^C with the 1,2,4-trichlorobenzene solvent. The X-ray diffraction (XRD) analysis was performed using a Rigaku Ultima IV powder X-ray diffractometer (Rigaku, Japan) at room temperature, utilizing Cu Kα radiation (45 kV, 40 mA, wavelength 1.540538 Å). The measurement range was set from 5° to 80°, with a scanning speed of 2°·min⁻¹. The Raman spectra were recorded by Raman spectroscopy (Dxr2xi, Thermo Fisher Scientific, USA) with a 532 nm laser excitation source. The FTIR spectra of TABQ-DHBQ and TAPT-DHBQ samples were obtained using a Nicolet iS50 FTIR spectrometer (Thermo Fisher Scientific, USA), with the samples prepared as KBr pellets. Electrode samples were analyzed in attenuated total reflection (ATR) mode, within a spectral range of 400–4000 cm⁻¹. Scanning electron microscopy (SEM) images and energy-dispersive X-ray spectroscopy (EDX) data were acquired using a Helios 5 CX microscope (Thermo Fisher Scientific, USA). Transmission electron microscopy (TEM) images were acquired using a JEM-F200 microscope (JEOL, Japan). Thermogravimetric analysis (TGA) was performed with a HITACHI STA200 TGA analyzer (Hitachi Ltd., Japan). The successful synthesis of monomers and polymers was confirmed by nuclear magnetic resonance (NMR) spectroscopy, conducted using an Ascend Aeon900 spectrometer (Bruker, Germany). X-ray photoelectron spectroscopy (XPS) was carried out with a K-ALPHA spectrometer from Thermo Fisher Scientific, USA.

1. **Electrochemical Measurements**

The cathodes were prepared by mixing TABQ-DHBQ or TAPT-DHBQ (60 wt%), Super P (30 wt%), and polyvinylidene fluoride (PVDF, 10 wt%) in N-methylpyrrolidone (NMP) to form a homogeneous slurry. The mixture was then uniformly coated onto graphite paper (0.1 mm thickness) using a vacuum adsorption coater (ZY-TB-X) and dried under vacuum at 60°C for 12 hours. The cathode loading mass was approximately 1 mg cm⁻². CR2032 coin-type cells were assembled to investigate the electrochemical performance of the two polymers, with Zn foil as the anode, 2 M ZnSO₄ aqueous solution or 2 M ZnSO₄ + 0.2 M ZnI_2_ aqueous solution as electrolyte, and glass fiber (Whatman GF/D) as the separator. Cyclic voltammetry (CV) tests were conducted using an electrochemical workstation (CHI760E). The energy storage performance of the cells was evaluated with a LAND multichannel battery test system (LAND CT3002A). Additionally, the galvanostatic intermittent titration technique (GITT) was applied using the LAND multichannel battery test system (LAND CT3002A) to analyze the diffusion kinetics at a current density of 100 mA g⁻¹, with a charge/discharge interval time of 10 minutes for each step.

1. **Computation method**

The equation for calculating the theoretical capacity is given by:

$$C_{T}=\frac{n\times F}{3.6\times M_{W}}$$

Where $\mathbf{C}_{\boldsymbol{T}}$ represents the theoretical specific capacity, $\boldsymbol{F}$ is the Faraday constant (96485 C mol^-1^), $\boldsymbol{n}$ is the number of transferred electrons, and $\boldsymbol{M}_{\boldsymbol{W}}$ is the molecular weight of the compound.^[1]^

The contributions of diffusion-controlled and capacitive effects can be quantified using the Trasatti analysis method. In cyclic voltammetry (CV) tests, the relationship between the peak current and the scan rate is commonly described by the power law:

$$i=av^{b}$$

Where $\mathbf{a}$ and $\boldsymbol{b}$ are adjustable parameters, and the value of $\boldsymbol{b}$ can be determined from the slope of the plot of log$\boldsymbol{i}$ versus log$\boldsymbol{v}$. A $\boldsymbol{b}$-value of 0.5 indicates that the current is mainly controlled by the insertion process, while a $\boldsymbol{b}$-value of 1 suggests that the capacitive effect dominates.

To further quantify the contributions of capacitive and diffusion-controlled effects to the total current response, the following equation can be used to separate these two contributions:

$$i(V)=k_{1}v+k_{2}v^{1/2}$$

Where $\boldsymbol{i}\mathbf{(}\boldsymbol{V}\mathbf{)}$ represents the total current response at a given potential, $\boldsymbol{k}_{\mathbf{1}}\boldsymbol{v}$ corresponds to the current arising from capacitive effects, and $\boldsymbol{k}_{\mathbf{2}}\boldsymbol{v}^{\mathbf{1/2}}$ accounts for the current due to the diffusion-controlled insertion process. This equation can be rearranged as:

$$\frac{i(V)}{v^{1/2}}=k_{1}v^{1/2}+k_{2}$$

By plotting $\frac{\boldsymbol{i}\mathbf{(}\boldsymbol{V}\mathbf{)}}{\boldsymbol{v}^{\mathbf{1/2}}}$ against $\boldsymbol{v}^{\mathbf{1}/\mathbf{2}}$, the values of $\boldsymbol{k}_{\mathbf{1}}$ (slope) and $\boldsymbol{k}_{\mathbf{2}}$​ (intercept) can be obtained from the straight line. This allows for the quantification of the contributions of surface capacitance and diffusion to the total current response at a specific voltage.^[2]^

The chemical diffusion coefficient was obtained as follows:

$$D_{\mathrm{Zn}^{2+}}=\frac{4}{\pi\tau}\left( \frac{n_{M}V_{M}}{S} \right)^{2}\left[ \frac{\Delta E_{s}}{\Delta E_{\tau}} \right]^{2}$$

Here, $\boldsymbol{\tau}$ represents the constant pulse time (10 min), $\boldsymbol{n}_{\boldsymbol{M}}$ and **V_M_** are the number of moles of the electrode and molar volume, respectively, $\boldsymbol{S}$ is the electrode–electrolyte interface area, $\boldsymbol{\Delta}\boldsymbol{E}_{\boldsymbol{s}}$ is the change of steady-state voltage during a single-step GITT experiment, and $\boldsymbol{\Delta}\boldsymbol{E}_{\boldsymbol{\tau}}$ stands for the total change of cell voltage during a constant current pulse s of a single-step GITT experiment regardless of the IR-drop.^[3]^

The optimized and energy calculation of the two polymers and the possible discharge configuration were simulated using Density Functional Theory (DFT) with the Gaussian 09W software package. The B3LYP functional and the 6-31+g (d) basis set were used for the calculations, which included determining the lowest unoccupied molecular orbital (LUMO), the highest occupied molecular orbital (HOMO), and the molecular electrostatic potential (ESP) distribution. Geometries of the two polymers and their complexes with I_2_, I_3_^-^ and I^-^ were optimized using the dispersion corrected hybrid functional B3LYP-D3 with the lanl2dz basis set for iodine atoms and the 6-311+G (2d,p) basis set for rest of the atoms.

MD simulations were determined using Materials Studio package. The forced field parameters for all particles were obtained from Universal force fields. The TIP3P water model was used for H_2_O. The time step was 1 fs. The Van der Waals force and electrostatic energy were calculated using Atom-based and Ewald summation methods, respectively, with the cutoff distance of 15.5 Å. The standard periodic boundary condition was used in all simulations. Geometry optimization of the initial structure was performed based on the smart algorithm to minimize the energy of the system for the subsequent MD simulation. Following relaxation, the system was simulated for 2 ns under canonical ensemble (NVT)for data collection and statistical analyses. The periodic boundary cell length(Å) was 45.00(x), 25.00(y), 66.00(z) for TABQ-DHBQ and 60.00(x), 25.00(y), 55.00(z) for TAPT-DHBQ. And the solution model contained one-layer armchair' graphene sheet with 40 ZnSO4 molecules, and 1110 H_2_O molecules.


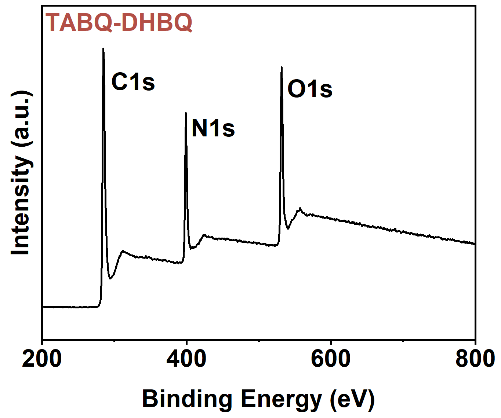


**Figure S1.** XPS spectrum of TABQ-DHBQ polymer.


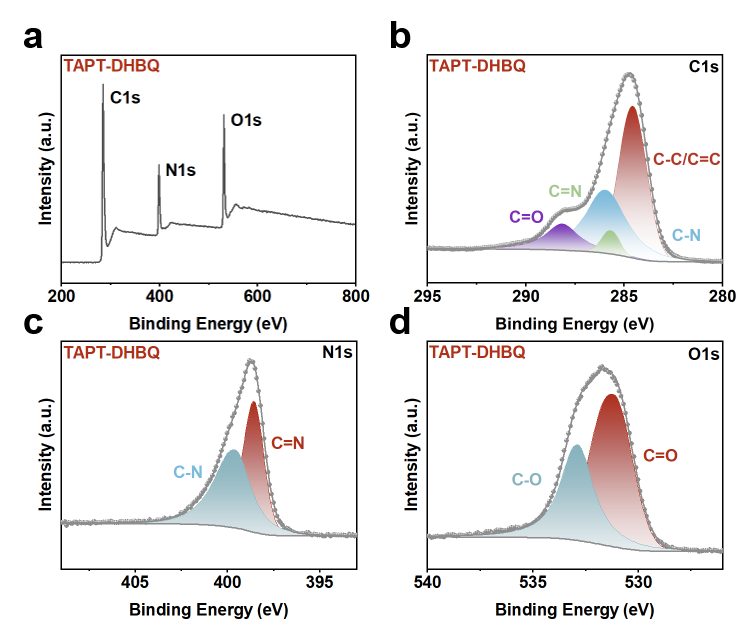


**Figure S2.** XPS spectra of TAPT-DHBQ polymer. (a) full spectrum, (b) C1s, (c) N1s, and (d) O1s.

**
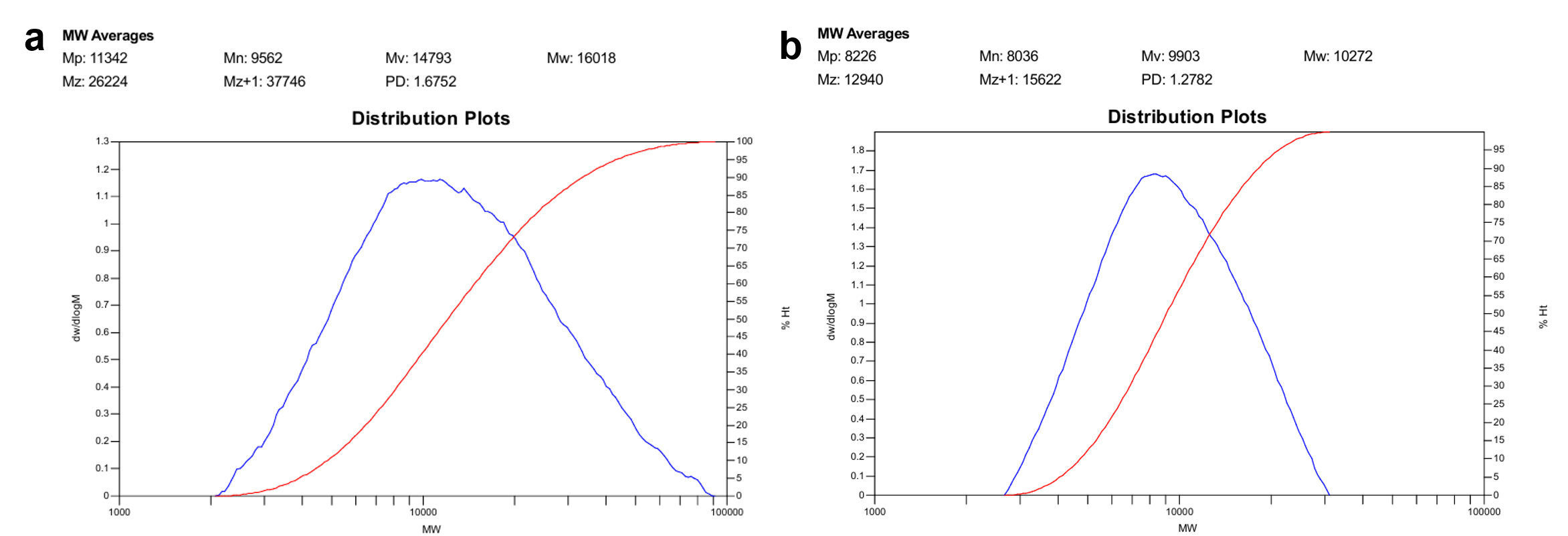
**

**Figure S3.** GPC report of TABQ-DHBQ polymer (a) and TAPT-DHBA polymer (b).


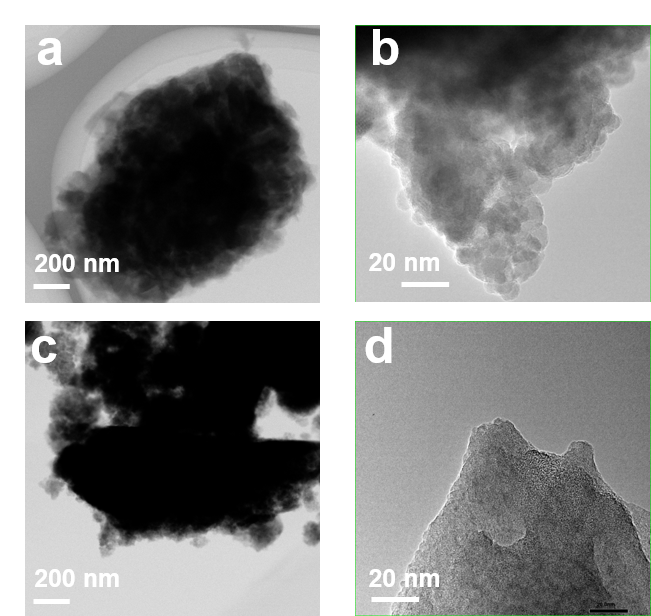


**Figure S4.** TEM images of TABQ-DHBQ polymer (a, b) and TAPT-DHBQ polymer (c, d).


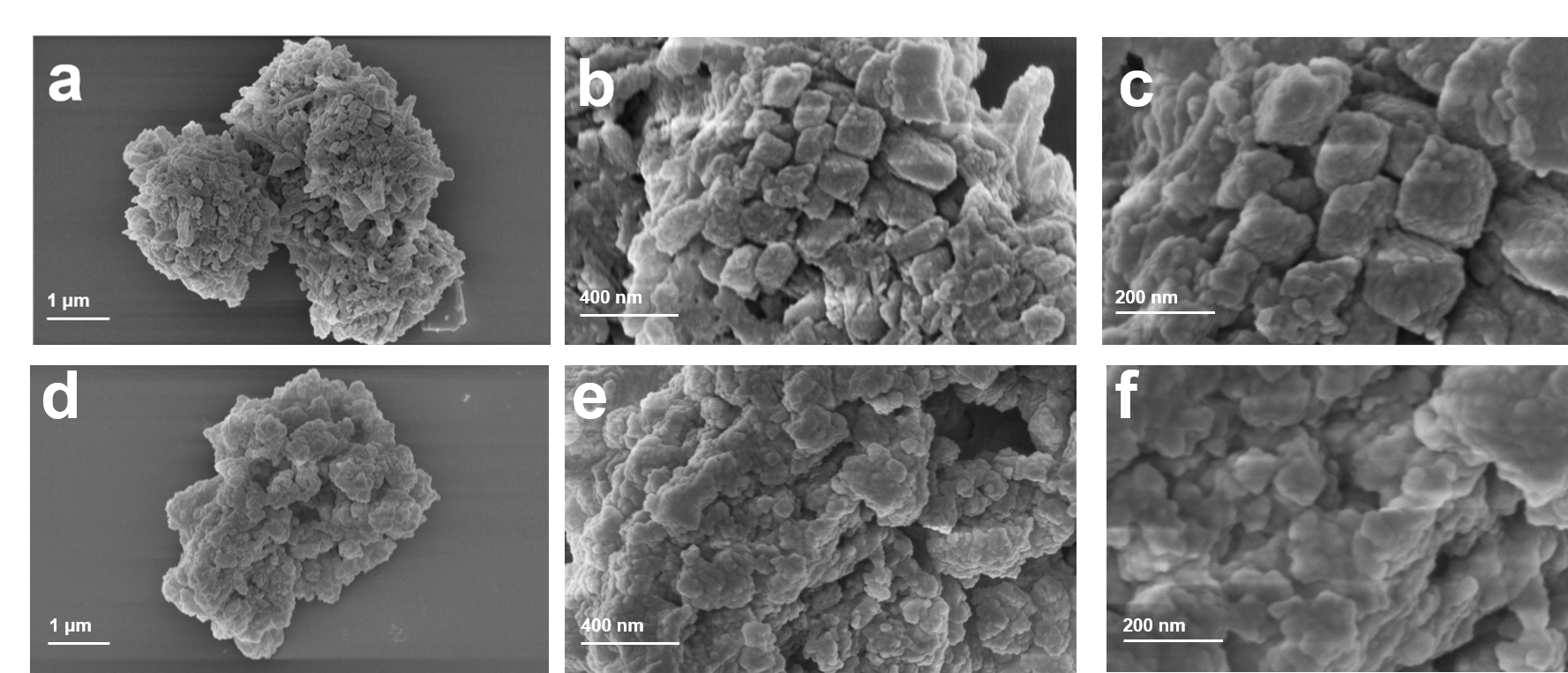


**Figure S5.** SEM images of TABQ-DHBQ (a-c) and TAPT-DHBQ (d-e) polymers.


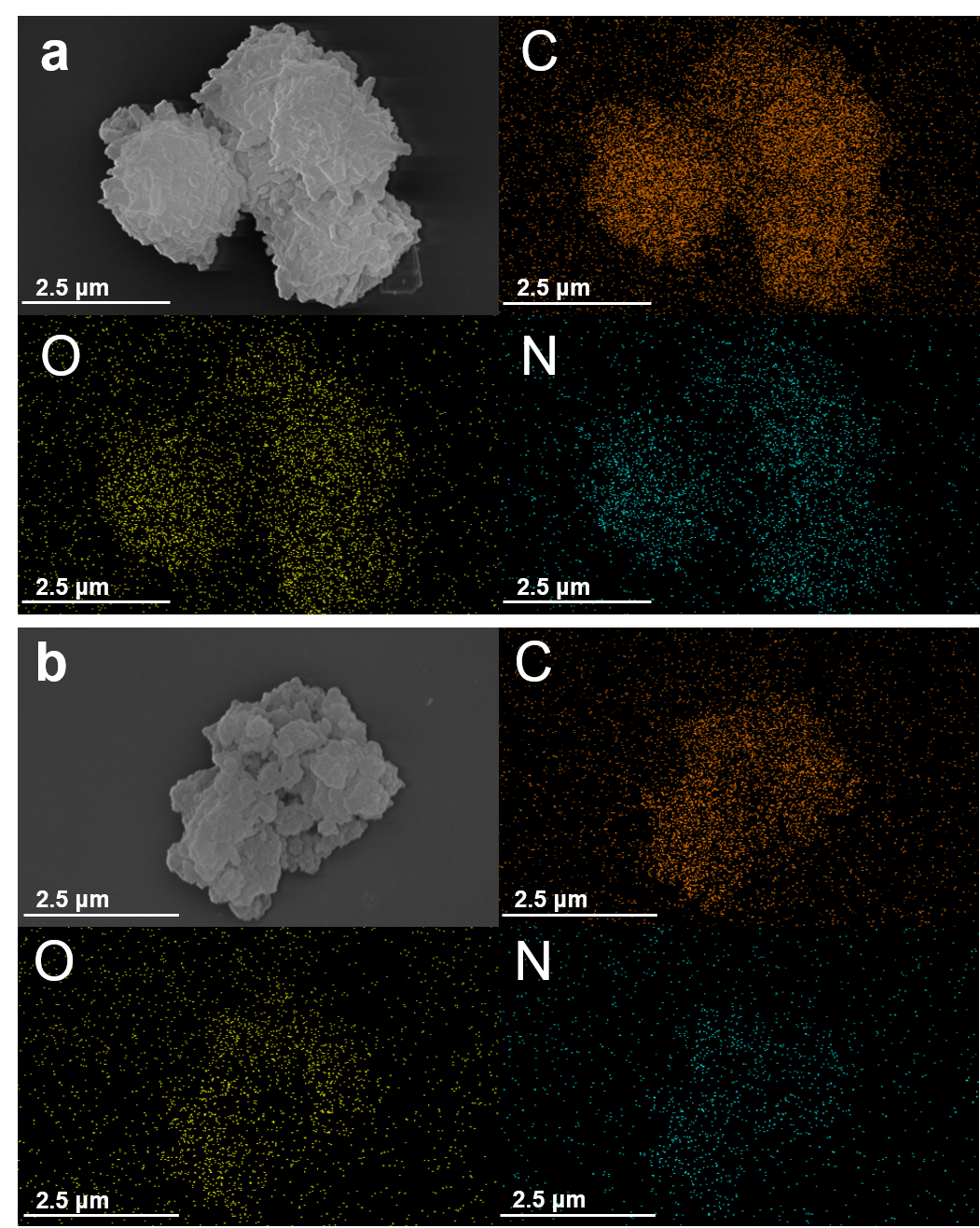


**Figure S6.** EDX element mappings of TABQ-DHBQ (a) and TAPT-DHBQ (b) polymers.


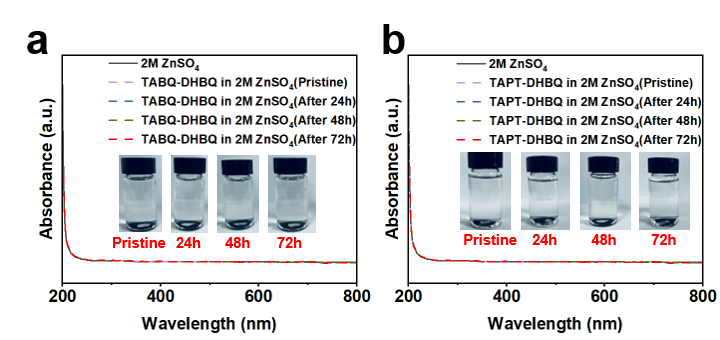


**Figure S7.** UV-vis spectra of bare aqueous electrolyte (2 M ZnSO_4_) and the electrolyte immersed by TABQ-DHBQ (a) or TAPT-DHBQ (b) cathodes for 72 h.


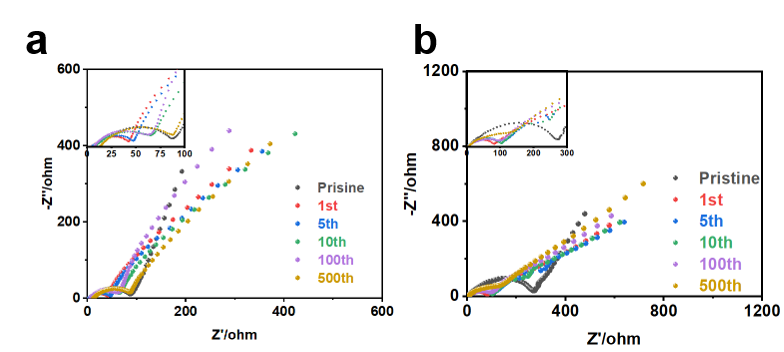


**Figure S8.** EIS analysis of TABQ-DHBQ (a) and TAPT-DHBQ (b) cathodes.

**
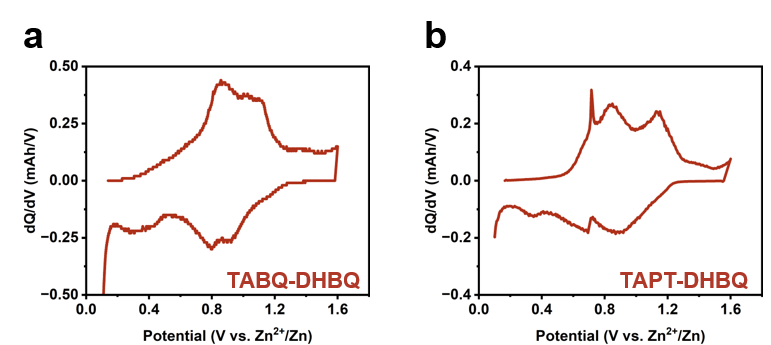
**

**Figure S9.** dQ/dV curves of Zn||TABQ-DHBQ batteries (a) and Zn||TAPT-DHBQ batteries (b).


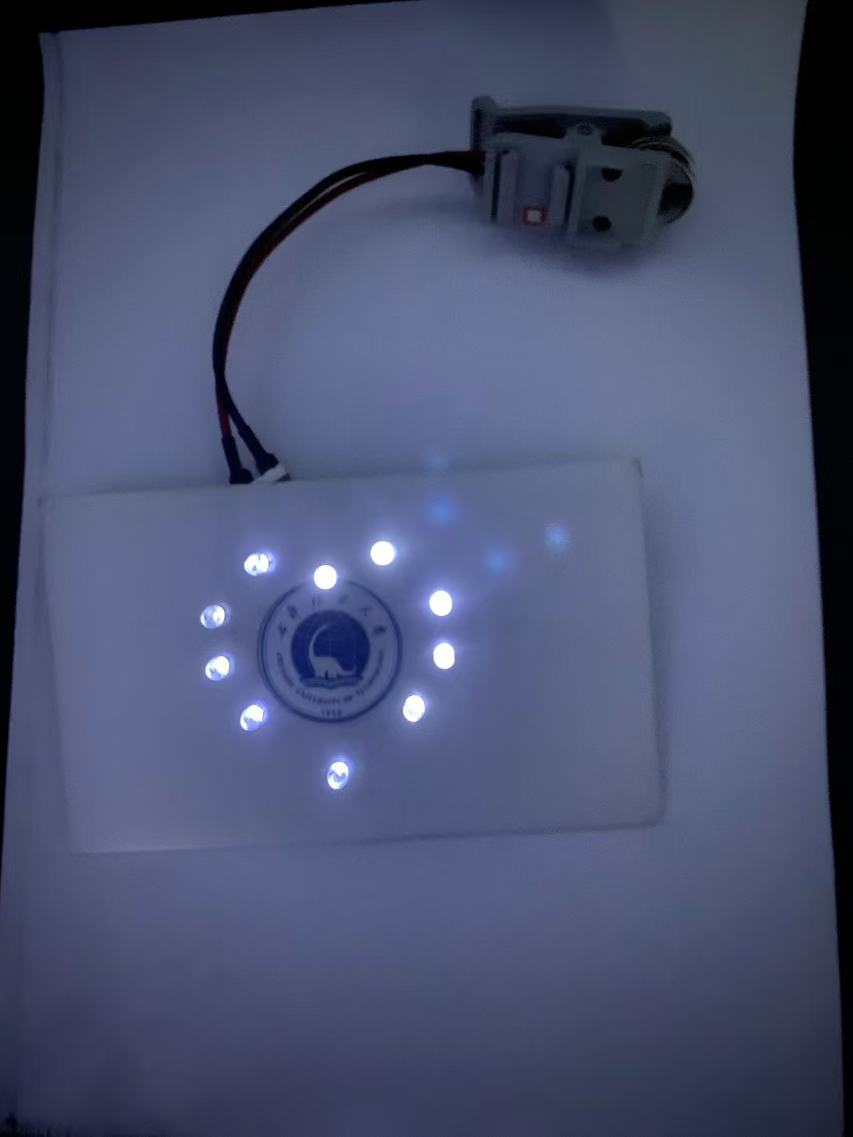


**Figure S10.** The digital photo of LED pattern lit up with three batteries based on TABQ-DHBQ cathodes.


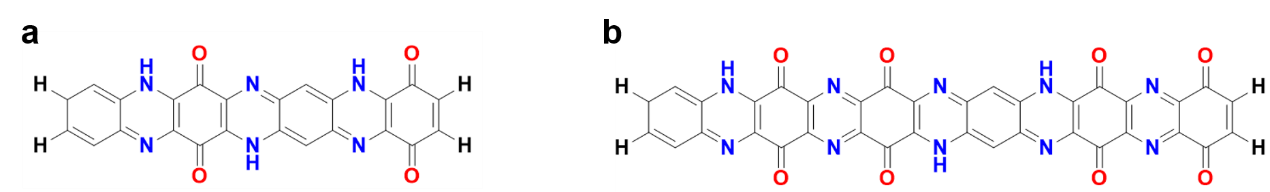


**Figure S11.** The structure units of TABQ-DHBQ (a) and TAPT-DHBQ (b) polymers used for simulation calculations.


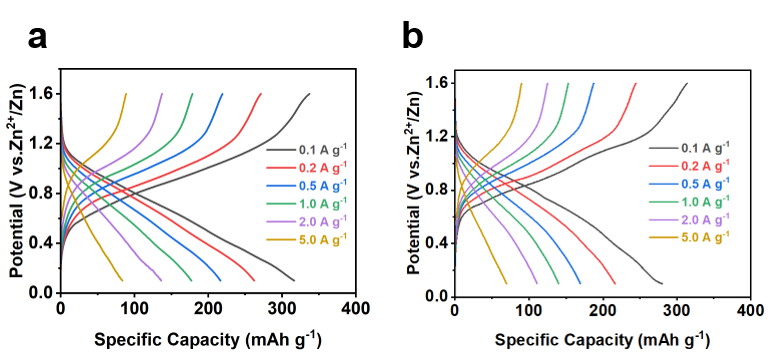


**Figure S12.** GCD curves of TABQ-DHBQ (a) and TAPT-DHBQ (b) cathodes at different current densities.


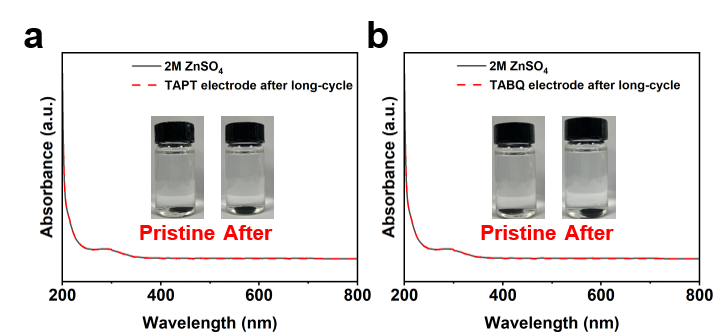


**Figure S13.** UV-vis spectra of bare aqueous electrolyte (2 M ZnSO_4_) and the electrolyte immersed by TABQ-DHBQ (h) or TAPT-DHBQ (i) cathodes after 2,000 charge/discharge process.

**
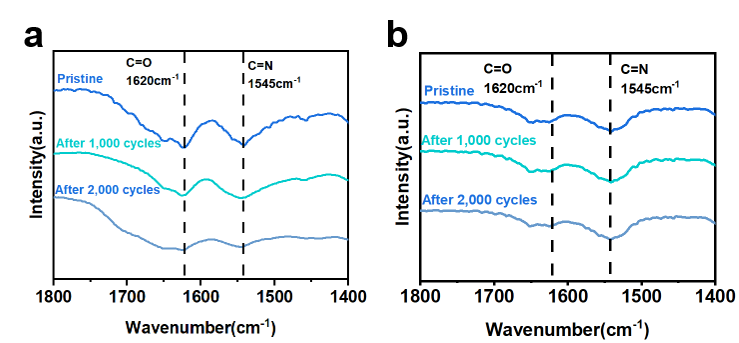
**

**Figure S14.** FTIR spectra of TABQ-DHBQ cathodes (a) and TAPT-DHBQ cathodes (b) in their initial state, after 1,000 charge/discharge cycles and after 2,000 charge/discharge cycles.


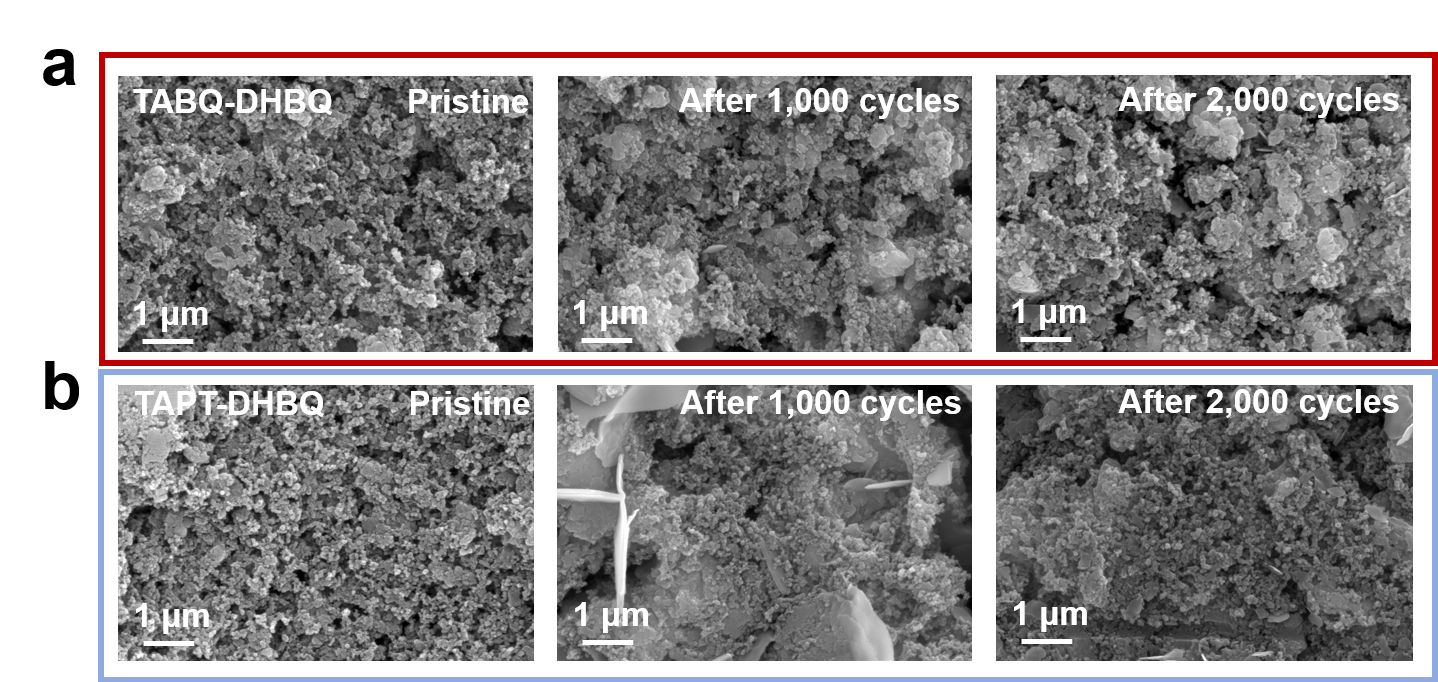


**Figure S15.** SEM images of TABQ-DHBQ cathodes (a) and TAPT-DHBQ cathodes (b) in their initial state, after 1,000 charge/discharge cycles and after 2,000 charge/discharge cycles.

**
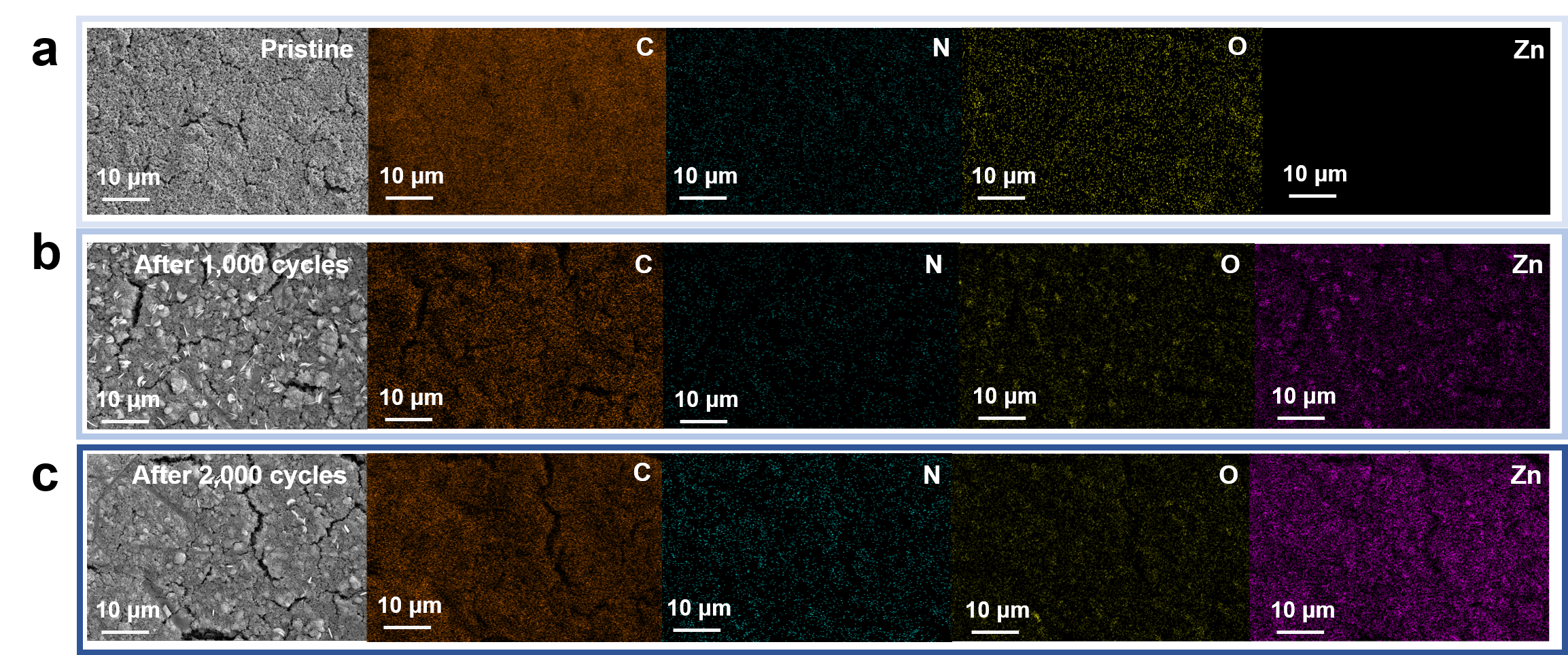
**

**Figure S16.** EDX mappings of TABQ-DHBQ cathodes in their initial state (a), after 1,000 charge/discharge cycles (b) and after 2,000 charge/discharge cycles (c).

**
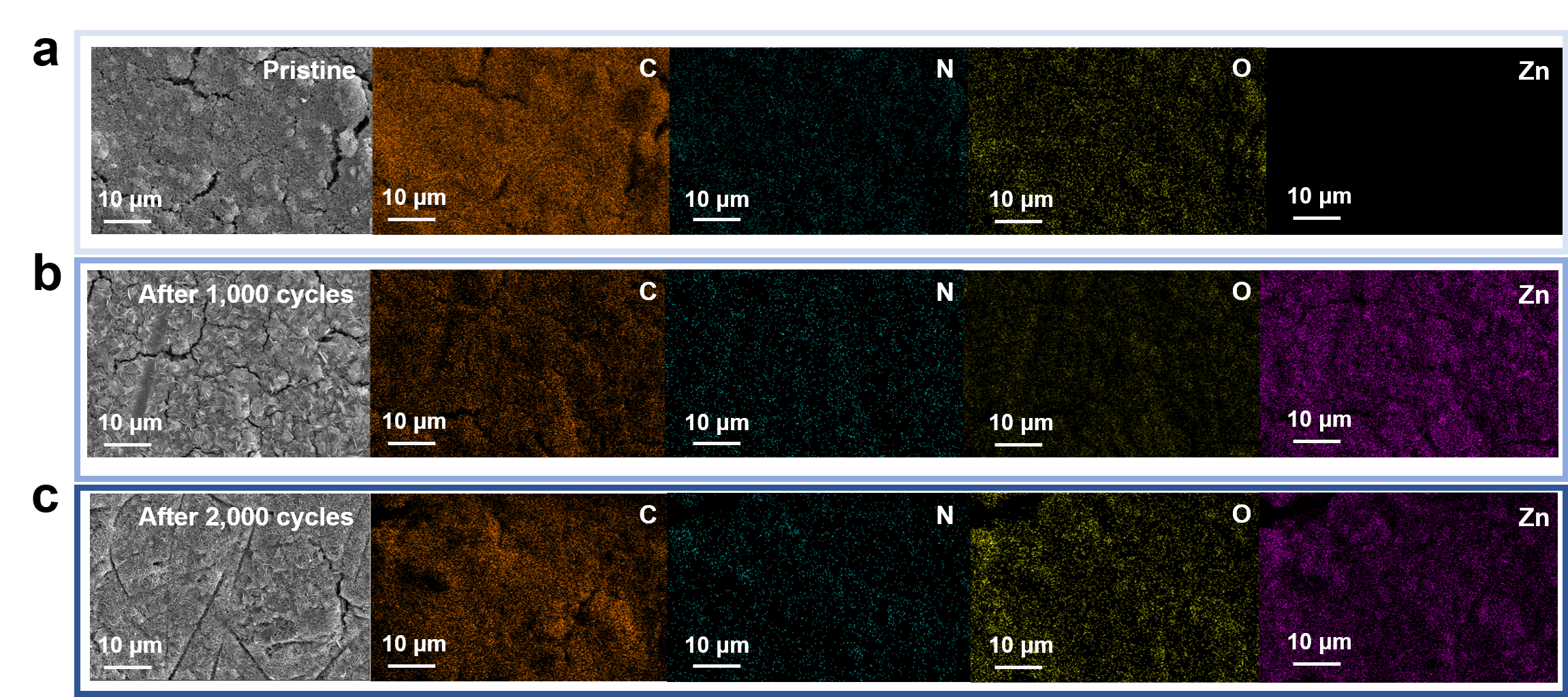
**

**Figure S17.** EDX mappings of TAPT-DHBQ cathodes in their initial state (a), after 1,000 charge/discharge cycles (b) and after 2,000 charge/discharge cycles (c).

**
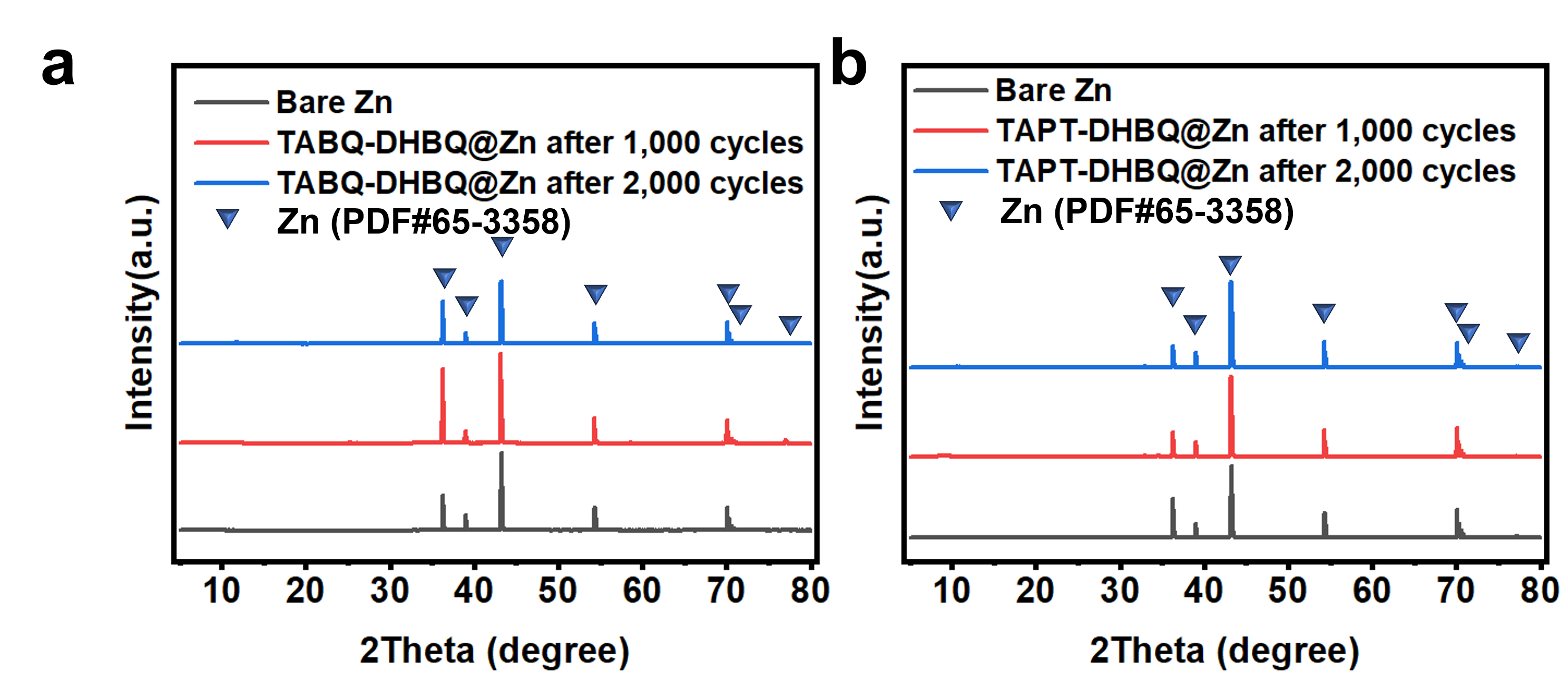
**

**Figure S18.** XRD patterns of zinc anode in Zn||TABQ-DHBQ battery (a) and Zn||TAPT-DHBQ battery (b) at their initial state, after 1,000 charge/discharge cycles and after 2,000 charge/discharge cycles.

**
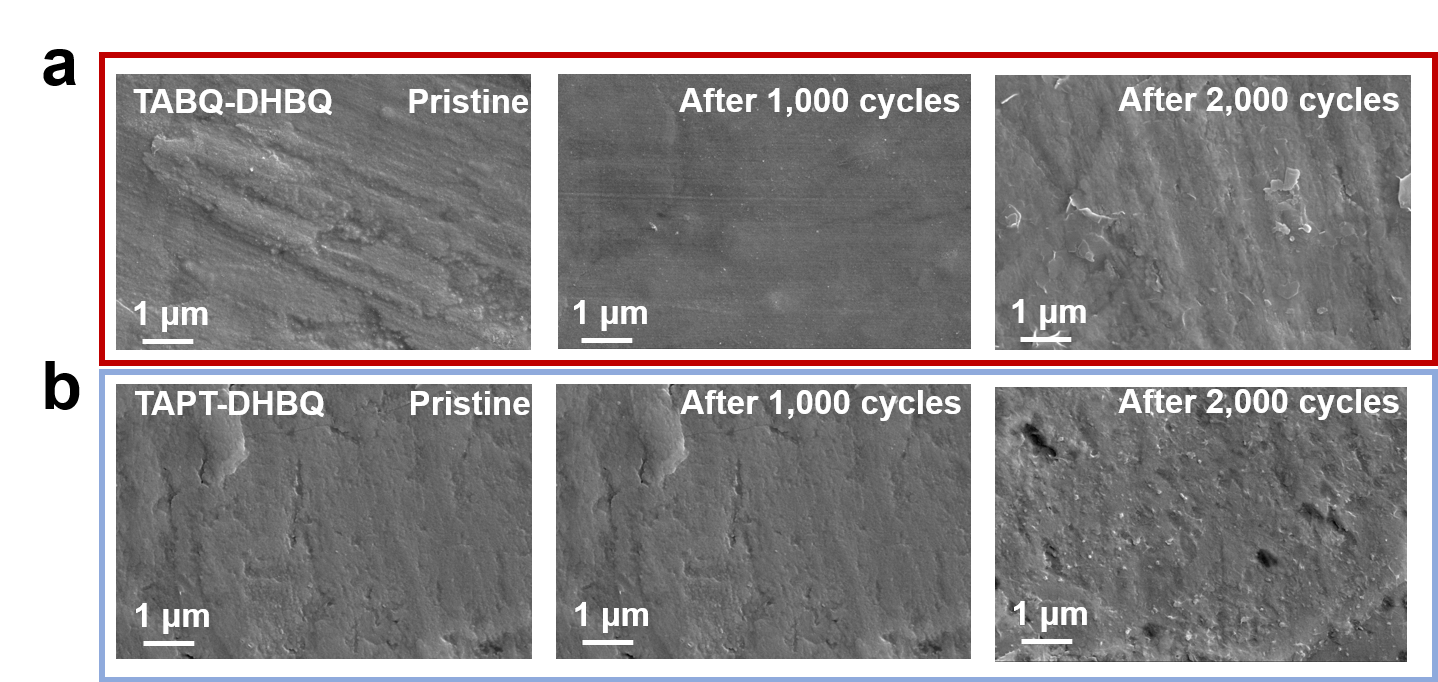
**

**Figure S19.** SEM images of zinc anode in Zn||TABQ-DHBQ battery (a) and Zn||TAPT-DHBQ battery (b) at their initial state, after 1,000 charge/discharge cycles and after 2,000 charge/discharge cycles.


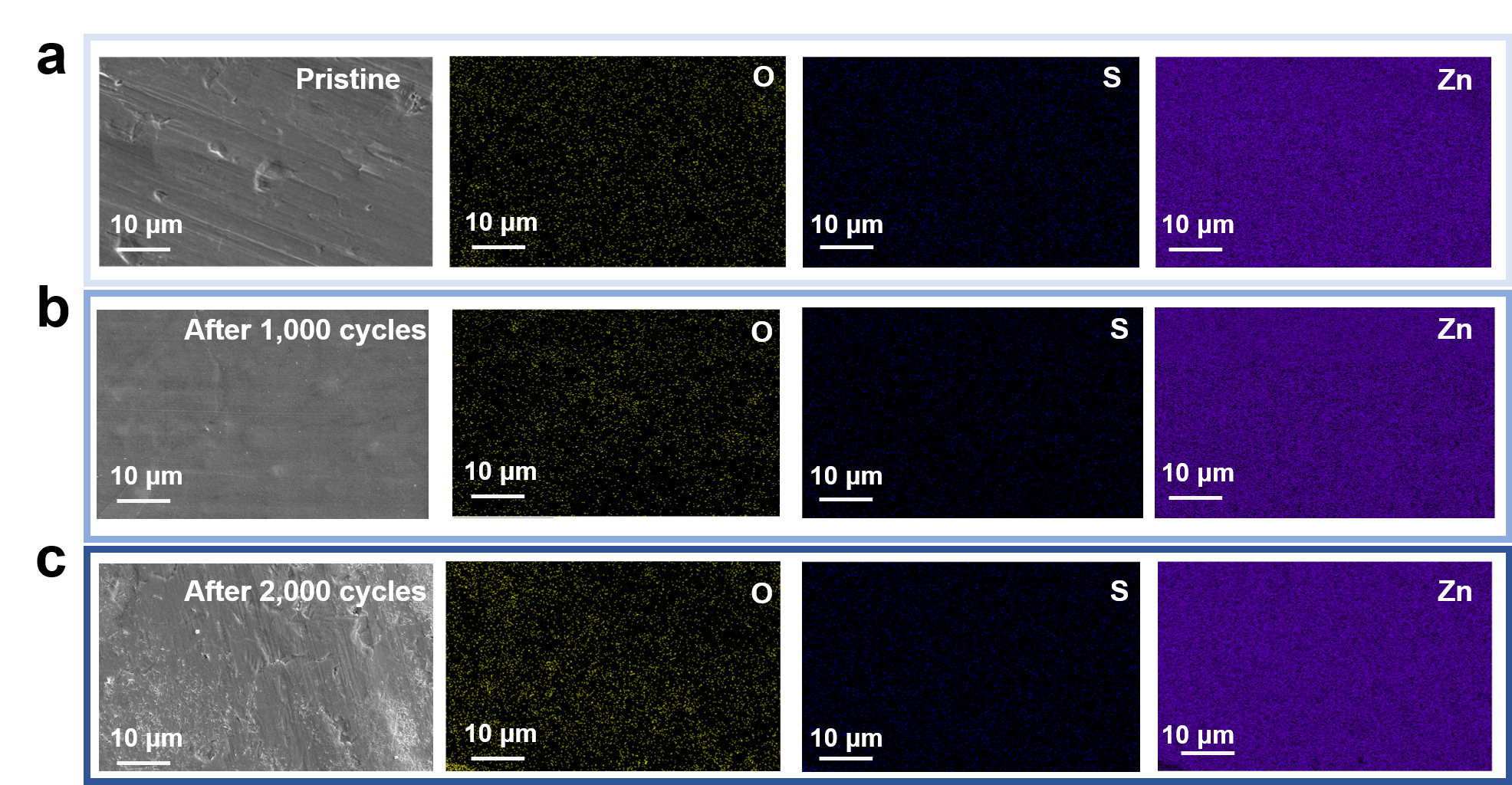


**Figure S20.** EDX mappings of zinc anode in Zn||TABQ-DHBQ battery at their initial state (a), after 1,000 charge/discharge cycles (b) and after 2,000 charge/discharge cycles (c).


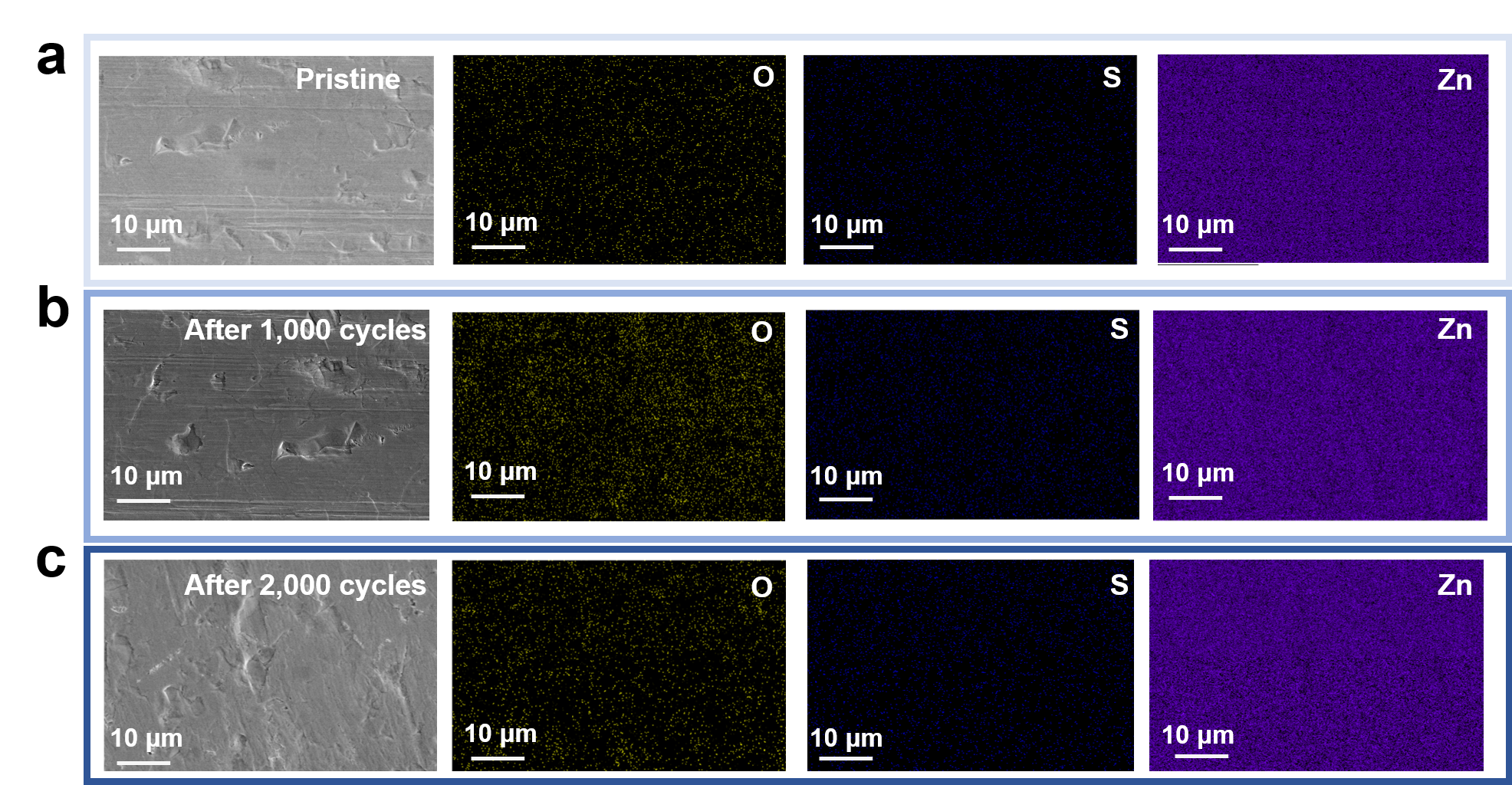


**Figure S21.** EDX mappings of zinc anode in Zn||TAPT-DHBQ battery at their initial state (a), after 1,000 charge/discharge cycles (b) and after 2,000 charge/discharge cycles (c).


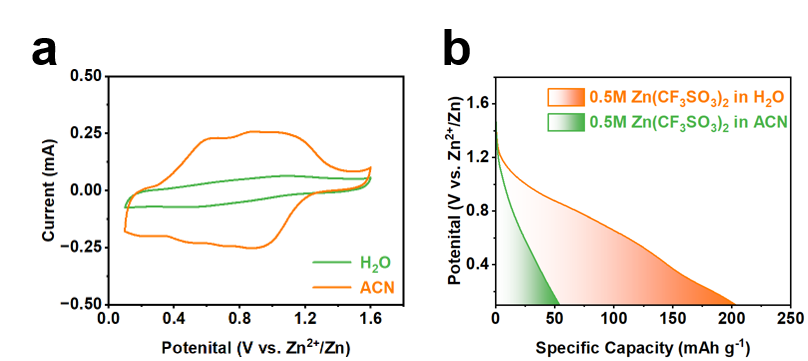


**Figure S22.** Electrochemical performance of TAPT-DHBQ electrodes in 0.5 M Zn(CF_3_SO_3_)_2_ electrolyte with either deionized water or anhydrous acetonitrile as the solvent. (a) CV curves. (b) GCD curves.

**
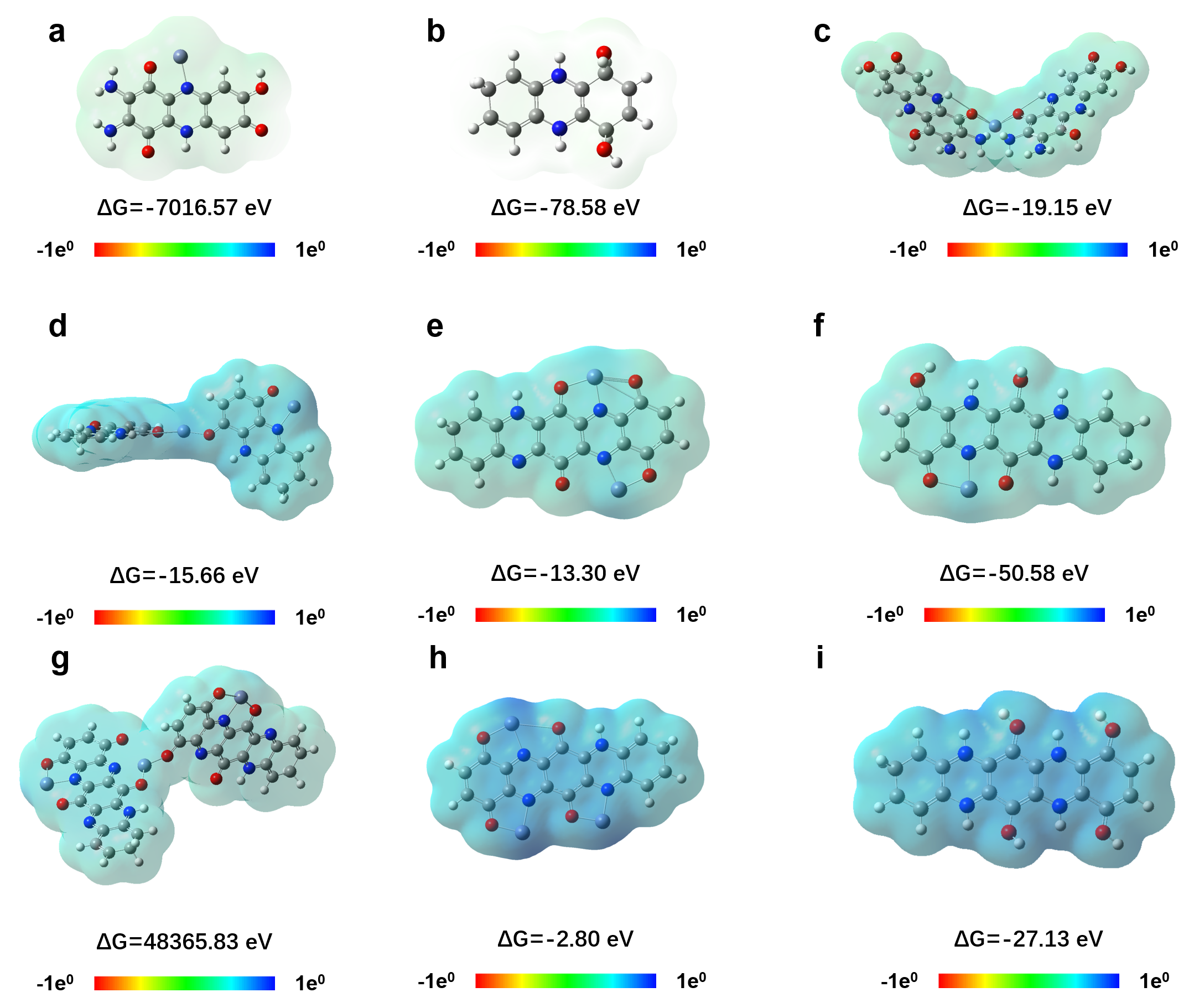
**

**Figure S23.** (a-d) The possible discharge configurations of TABQ-DHBQ polymers with their binding energy. (e-f) The possible discharge configurations of TAPT-DHBQ polymers with their binding energy.


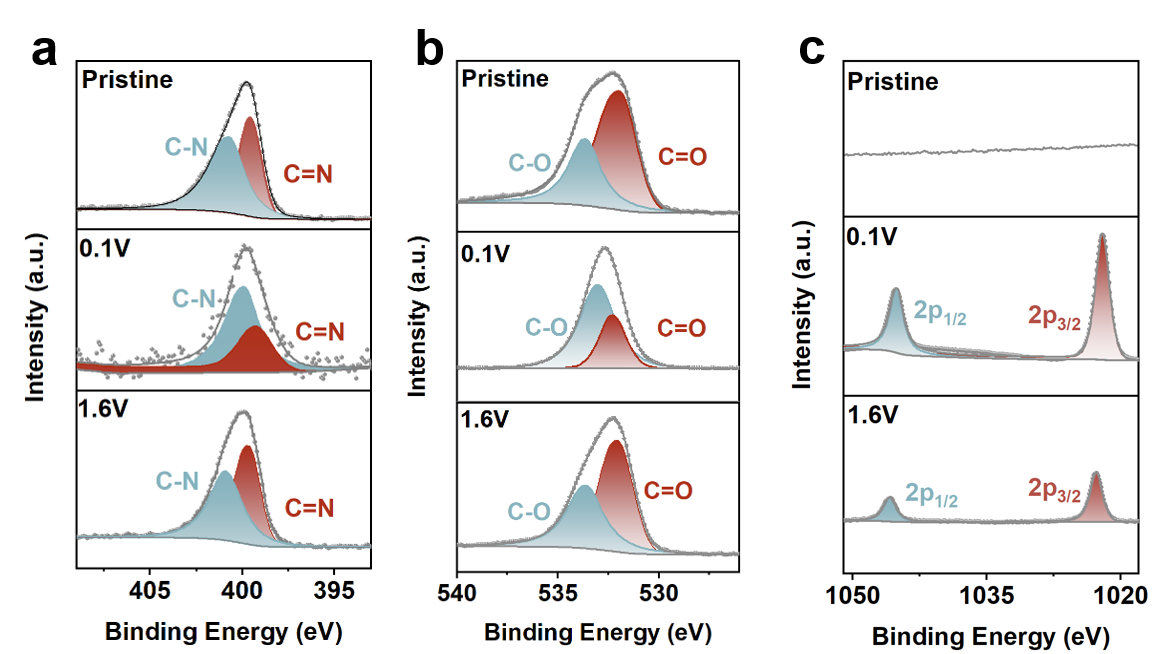


**Figure S24.** Ex-XPS spectra of TAPT-DHBQ for (a) N 1s, (b) O 1s, and (c) Zn 2p.


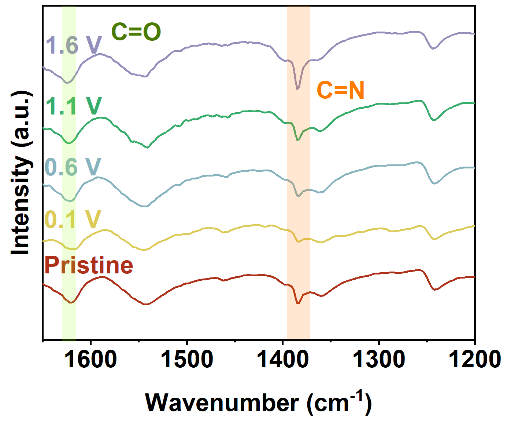


**Figure S25.** Ex-situ FTIR spectra of TAPT-DHBQ cathode at different charge/discharge states.


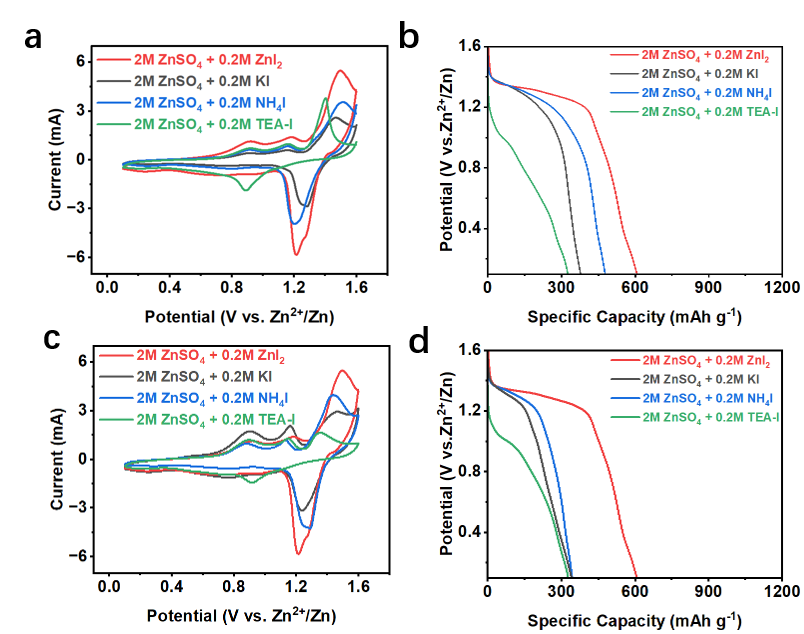


**Figure S26.** (a, b) The electrochemical performance of Zn||TABQ-DHBQ batteries based on different electrolyte additives. (c, d) The electrochemical performance of Zn||TAPT-DHBQ batteries based on different electrolyte additives. CV scan rate: 1 mV s^-1^, GCD charge/discharge current density: 1 A g^-1^.


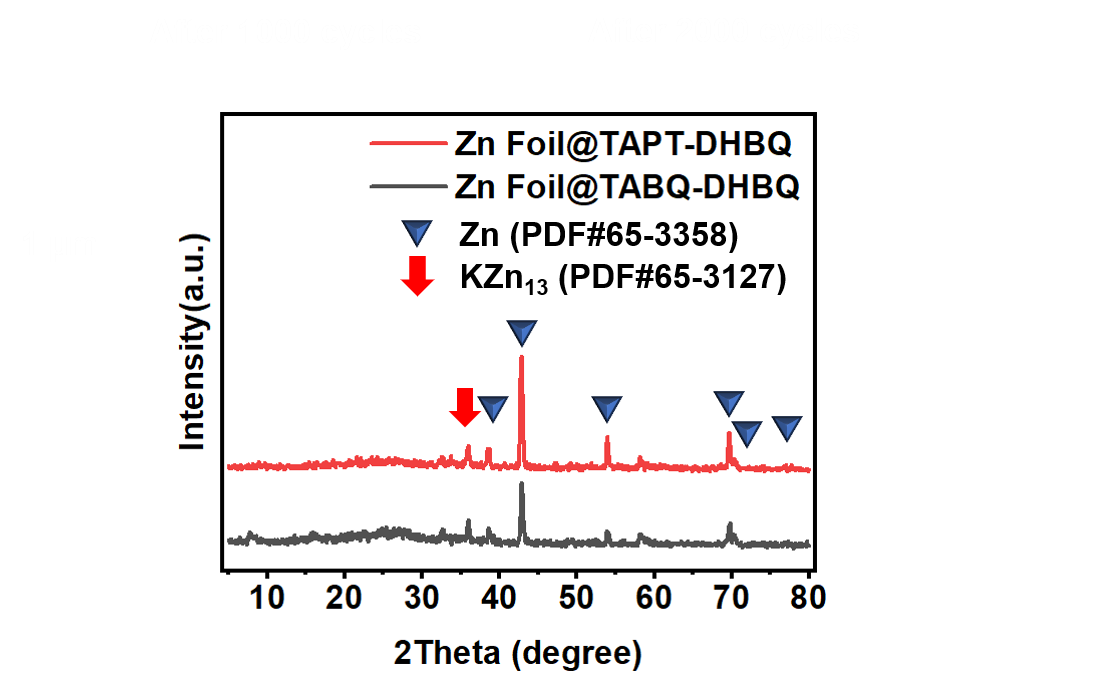


**Figure S27.** XRD patterns of zinc anode in AZIBs with 0.2 M KI electrolyte additive.


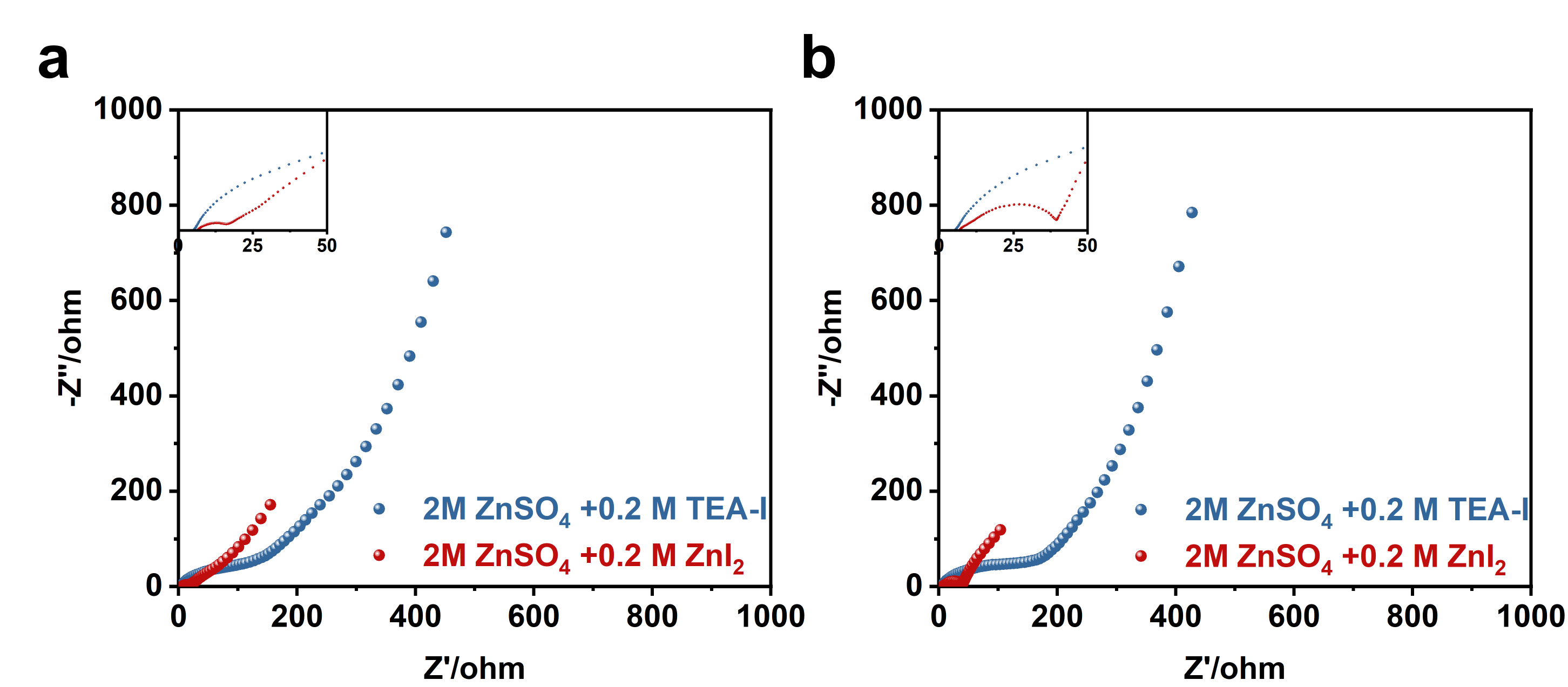


**Figure S28.** EIS curves of ZnI_2_ additive-based AZIBs and TEA-I additive-based AZIBs. (a) Zn||TABQ-DHBQ batteries. (b) Zn||TAPT-DHBQ batteries.


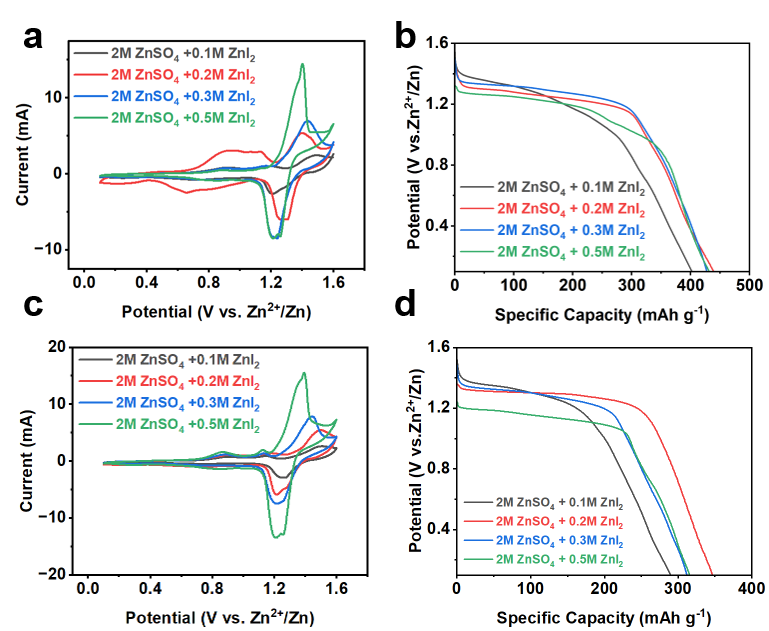


**Figure S29.** (a, b) The electrochemical performance of Zn||TABQ-DHBQ batteries with different concentration ZnI_2_ electrolyte additives. (c, d) The electrochemical performance of Zn||TAPT-DHBQ batteries with different concentration ZnI_2_ electrolyte additives. CV scan rate: 1 mV s^-1^, GCD charge/discharge current density: 5 A g^-1^.


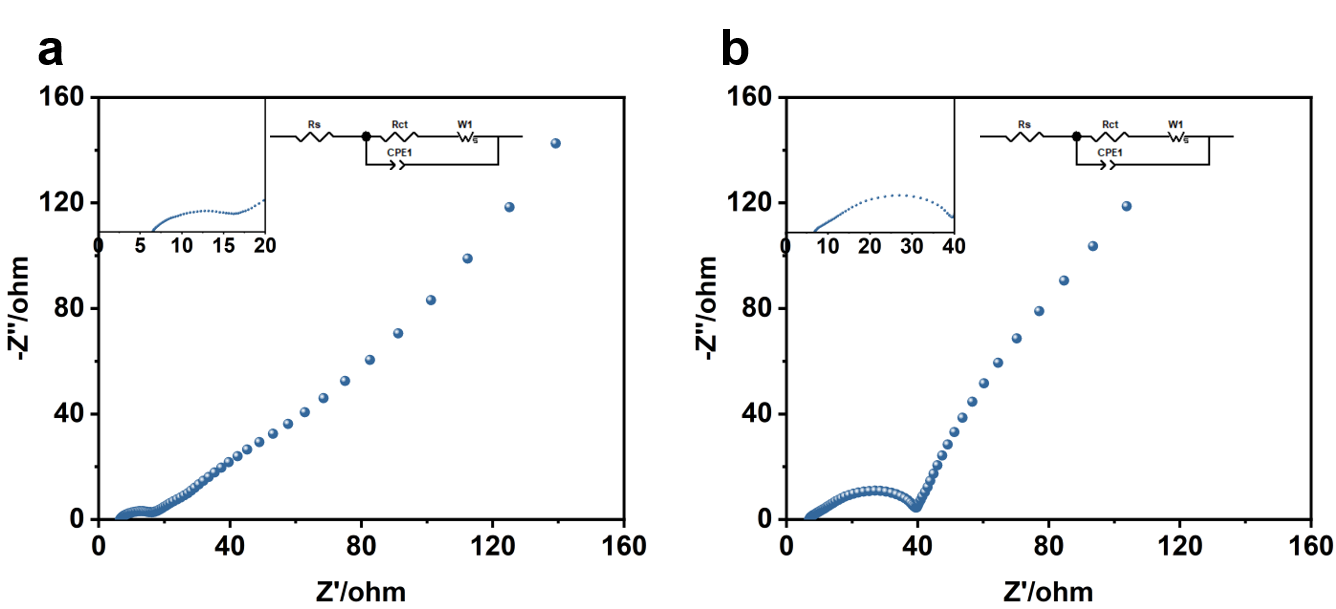


**Figure S30.** EIS analysis of TABQ-DHBQ cathode in 2 M ZnSO_4_ + 0.2 M ZnI_2_ (a) and 2 M ZnSO_4_ (b) electrolyte respectively.


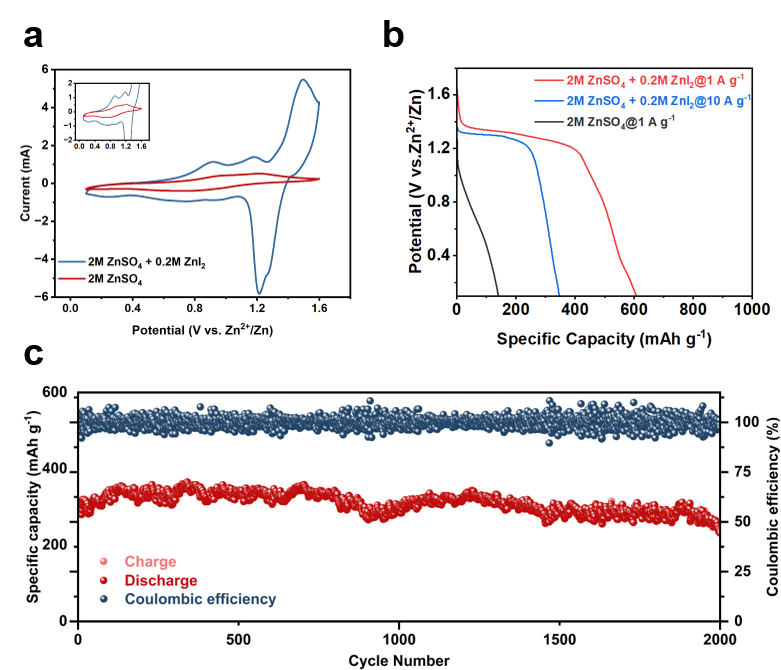


**Figure S31.** (a) The CV curves of Zn||TAPT-DHBQ batteries with or without ZnI_2_ electrolyte additive respectively at the scan rate of 1 mV s^–1^. (b) The GCD curves of Zn||TAPT-DHBQ batteries with or without ZnI_2_ electrolyte additive respectively at the current density of 1 A g^-1^. (c) Long-term cycling stability of Zn//TAPT-DHBQ batteries with ZnI_2_ electrolyte additive carried out at the current density of 10 A g^-1^.


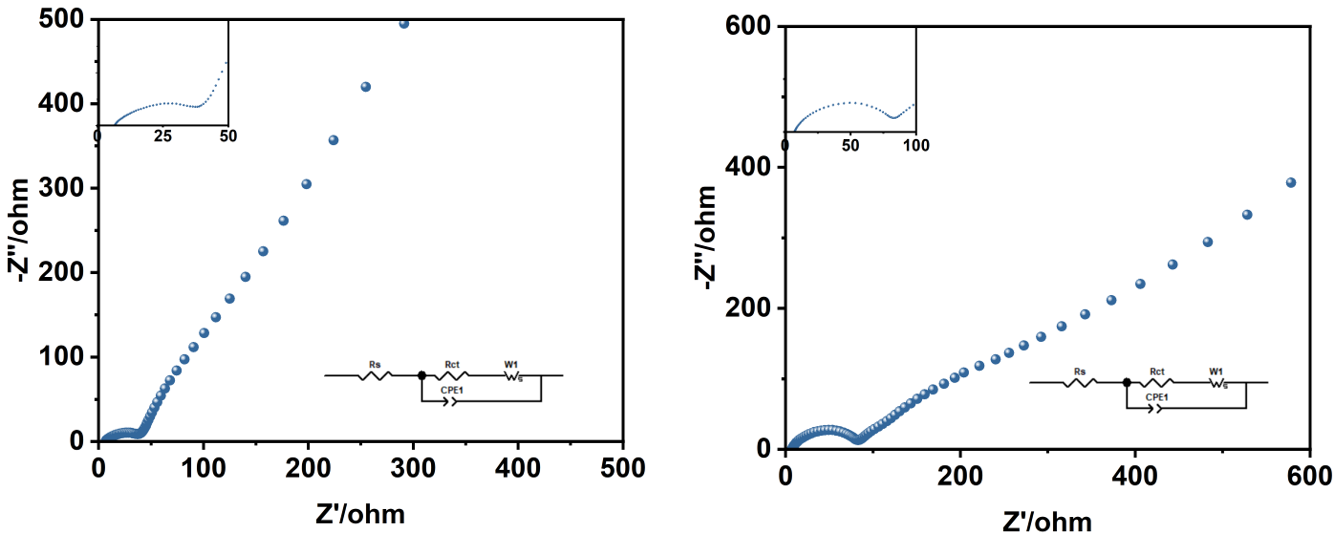


**Figure S32.** EIS analysis of TAPT-DHBQ cathode in 2 M ZnSO_4_ + 0.2 M ZnI_2_ (a) and 2 M ZnSO_4_ (b) electrolyte respectively.

**
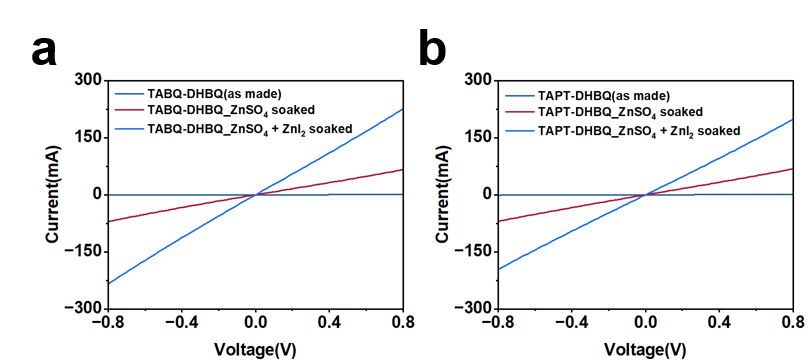
**

**Figure S33.** I-V curves of the bare two polymer cathodes, and the two polymer cathodes immersed in 2 M ZnSO_4_ electrolyte or 2 M ZnSO_4_ + 0.2 M ZnI_2_ electrolyte respectively.


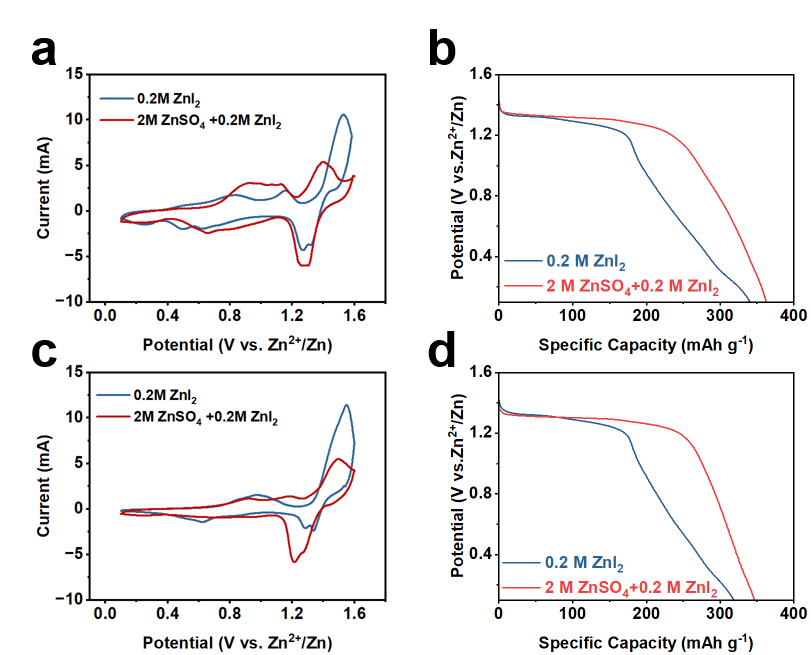


**Figure S34.** (a, b) The electrochemical performance of Zn||TABQ-DHBQ batteries with pure ZnI_2_ electrolyte or ZnSO_4_-ZnI_2_ mixed electrolyte. (c, d) The electrochemical performance of Zn||TAPT-DHBQ batteries with pure ZnI_2_ electrolyte or ZnSO_4_-ZnI_2_ mixed electrolyte. CV scan rate: 1 mV s^-1^, GCD charge/discharge current density: 10 A g^-1^.

**
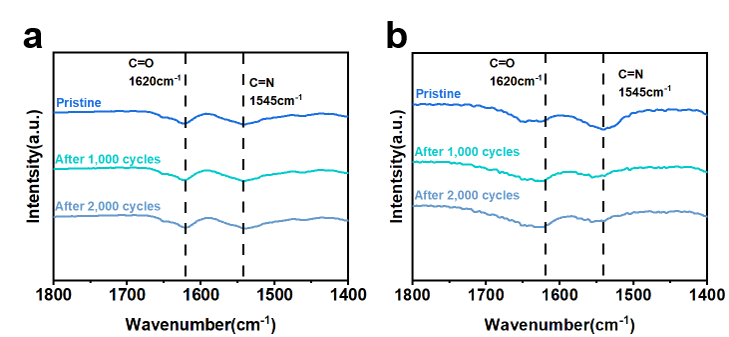
**

**Figure S35.** FTIR spectra of TABQ-DHBQ cathodes (a) and TAPT-DHBQ cathodes (b) in AZIBs with ZnI_2_ electrolyte additive at their initial state, after 1,000 charge/discharge cycles and after 2,000 charge/discharge cycles.


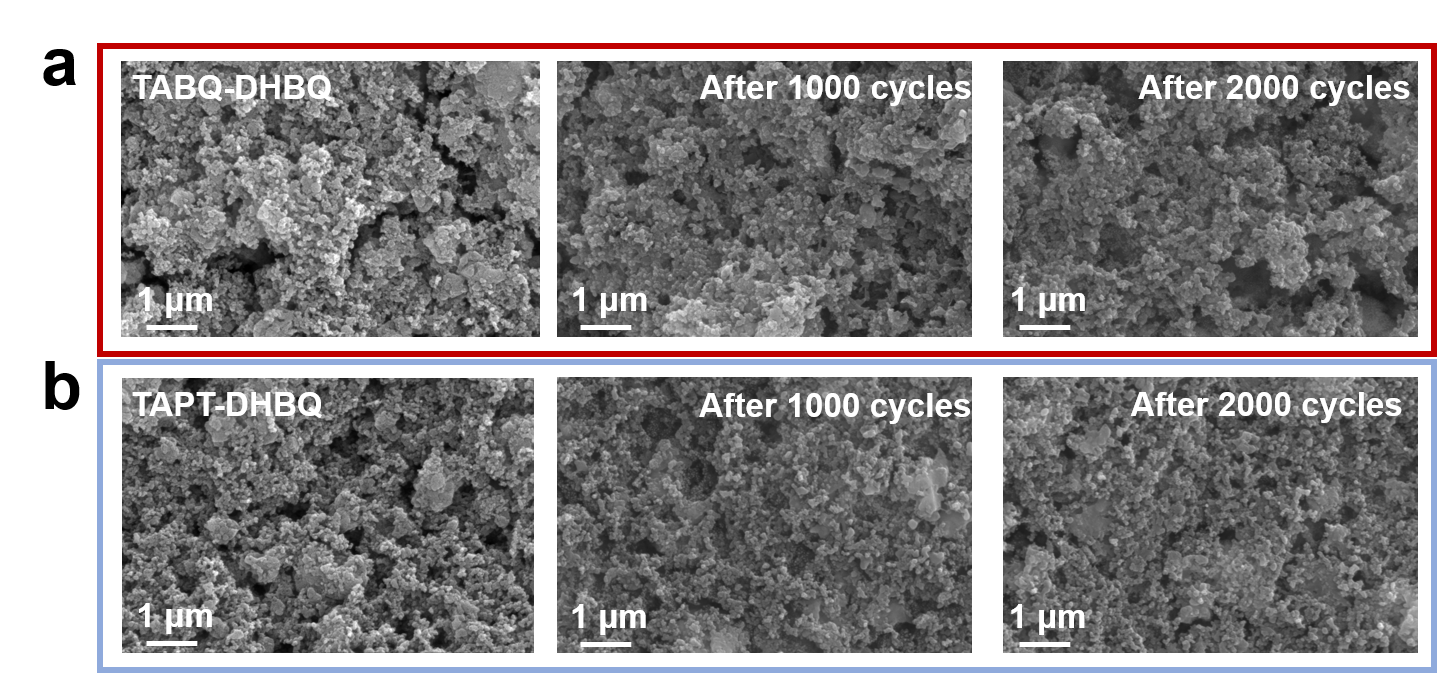


**Figure S36.** SEM images of TABQ-DHBQ cathodes (a) and TAPT-DHBQ cathodes (b) in AZIBs with ZnI_2_ electrolyte additive at their initial state, after 1,000 charge/discharge cycles and after 2,000 charge/discharge cycles.


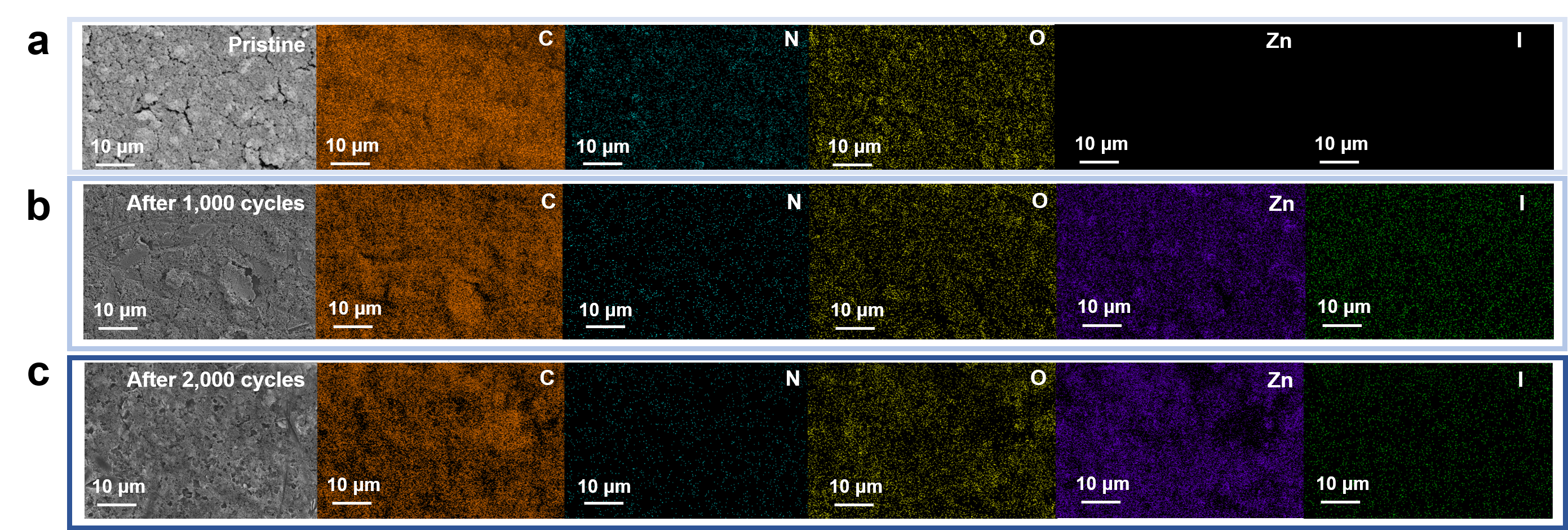


**Figure S37.** EDX mappings of TABQ-DHBQ cathodes in AZIBs with ZnI_2_ electrolyte additive at their initial state (a), after 1,000 charge/discharge cycles (b) and after 2,000 charge/discharge cycles (c).


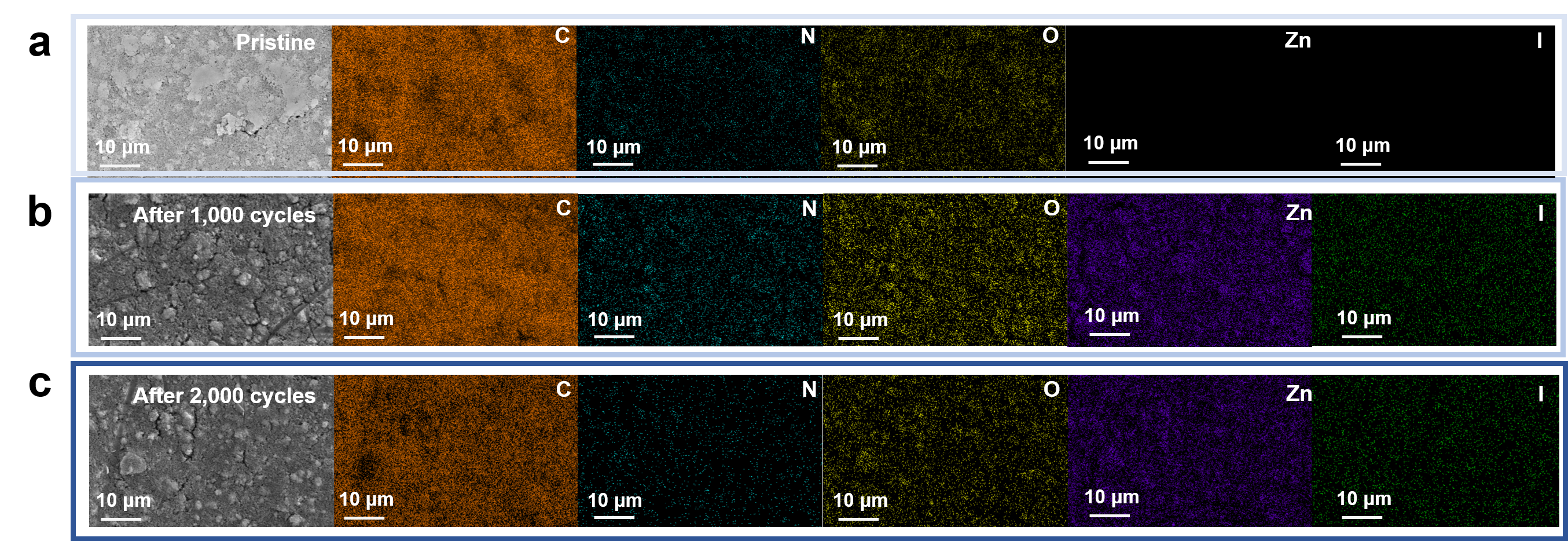


**Figure S38.** EDX mappings of TAPT-DHBQ cathodes in AZIBs with ZnI_2_ electrolyte additive at their initial state (a), after 1,000 charge/discharge cycles (b) and after 2,000 charge/discharge cycles (c).


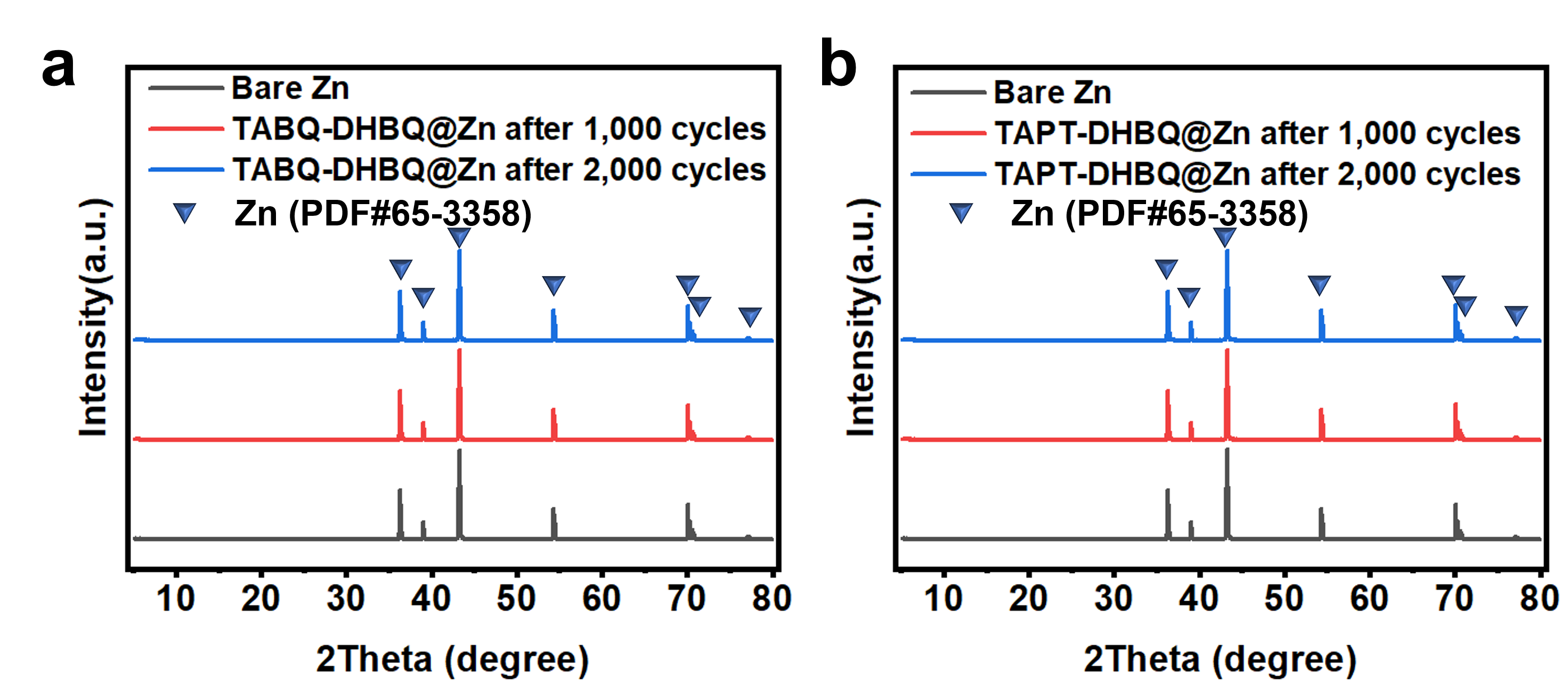


**Figure S39.** XRD patterns of zinc anode in Zn||TABQ-DHBQ batteries (a) and Zn||TAPT-DHBQ batteries (b) with ZnI_2_ electrolyte additive at their initial state, after 1,000 charge/discharge cycles and after 2,000 charge/discharge cycles.

**Table S1.** Comparison of utilization of active sites with other previously reported organic cathodes.

| **Organic cathodes** | **Electrolyte** | **Experimental Specific Capacity**  **(mAh g^-1^)** | **Theoretical Specific**  **Capacity**  **(mAh g^-1^)** | **Active Site Utilization**  **(%)** | **Energy Density**  **(Wh kg^-1^)** | **Ref.** |
| --- | --- | --- | --- | --- | --- | --- |
| PDBS | **2 M ZnSO_4_** | 260 | 289 | 89.9 | 157.1 | [4] |
| PBQS | **3 M Zn(OTf)_2_** | 203 | 350 | 58 | 193 | [5] |
| PCTB | **2 M ZnSO_4_** | 125 | 285 | 43 | 118.8 | [6] |
| PTO-4NH2Ph | **3 M ZnSO_4_** | 250 | 278 | 89.9 | -- | [7] |
| PTFHQ-A | **2 M Zn(OTf)_2_** | 306.24 | 319 | 96 | 212 | [1] |
| PPPA | **2 M Zn(OTf)_2_** | 210 | 442 | 48 | 209 | [8] |
| PDI-EDA/CB | **2 M ZnSO_4_** | 118 | 240 | 49 | -- | [9] |
| AOPS | **1 M Zn(OTf)_2_** | 170 | 216 | 79 | 135 | [10] |
| PBQ | **3.5 M Zn(ClO₄)₂** | 220 | 362 | 61 | -- | [11] |
| PTD-1 | **2 M ZnSO_4_** | 188.24 | 211.24 | 89.1 | 116.83 | [12] |
| DTT | **2 M ZnSO_4_** | 210.9 | 285 | 74 | 126.5 | [13] |
| BT-PTO-COF | **3 M Zn(OTf)_2_** | 225 | 343.61 | 65.5 | 92.4 | [14] |
| TAPT-DHBQ | **2 M ZnSO_4_** | 280 | 545.26 | 51.4 | 206.35 | This work |
| TABQ-DHBQ | **2 M ZnSO_4_** | 325 | 379.14 | 85.7 | 185.16 |  |
| TAPT-DHBQ | **2 M ZnSO_4_**  **+ 0.2 M ZnI_2_** | 607 | -- | -- | 670.70 |  |
| TABQ-DHBQ | **2 M ZnSO_4_**  **+ 0.2 M ZnI_2_** | 618 | -- | -- | 678.62 |  |

**References**

[1] Q. Q. Sun, T. Sun, J. Y. Du, Z. L. Xie, D. Y. Yang, G. Huang, H. M. Xie, X. B. Zhang, *Angew. Chem. Int. Ed.* **2023**, *62*, e202307365.

[2] F. Wan, L. Zhang, X. Wang, S. Bi, Z. Niu, J. Chen, *Adv. Funct. Mater.* **2018**, *28*, 1804975.

[3] M. Tang, Q. Zhu, P. Hu, L. Jiang, R. Liu, J. Wang, L. Cheng, X. Zhang, W. Chen, H. Wang, *Adv. Funct. Mater.* **2021**, *31*, 2102011.
